# Cut-like homeobox 1 (*CUX1*) tumor suppressor gene haploinsufficiency induces apoptosis evasion to sustain myeloid leukemia

**DOI:** 10.1038/s41467-021-22750-8

**Published:** 2021-04-30

**Authors:** Emmanuelle Supper, Saskia Rudat, Vivek Iyer, Alastair Droop, Kim Wong, Jean-François Spinella, Patrick Thomas, Guy Sauvageau, David J. Adams, Chi C. Wong

**Affiliations:** 1 Experimental Cancer Genetics, Wellcome Sanger Institute, Hinxton, UK; 2 The Leucegene Project at Institute for Research in Immunology and Cancer, Université de Montréal, 2950 Chemin de Polytechnique Pavillon, Marcelle-Coutu, Montréal, QC Canada; 3 Department of Haematology, Addenbrooke’s Hospital, Cambridge, UK

**Keywords:** Acute myeloid leukaemia, Myelodysplastic syndrome, Tumour-suppressor proteins

## Abstract

While oncogenes promote tumorigenesis, they also induce deleterious cellular stresses, such as apoptosis, that cancer cells must combat by coopting adaptive responses. Whether tumor suppressor gene haploinsufficiency leads to such phenomena and their mechanistic basis is unclear. Here, we demonstrate that elevated levels of the anti-apoptotic factor, CASP8 and FADD-like apoptosis regulator (CFLAR), promotes apoptosis evasion in acute myeloid leukemia (AML) cells haploinsufficient for the cut-like homeobox 1 (*CUX1*) transcription factor, whose loss is associated with dismal clinical prognosis. Genome-wide CRISPR/Cas9 screening identifies *CFLAR* as a selective, acquired vulnerability in *CUX1*-deficient AML, which can be mimicked therapeutically using inhibitor of apoptosis (IAP) antagonists in murine and human AML cells. Mechanistically, *CUX1* deficiency directly alleviates CUX1 repression of the *CFLAR* promoter to drive *CFLAR* expression and leukemia survival. These data establish how haploinsufficiency of a tumor suppressor is sufficient to induce advantageous anti-apoptosis cell survival pathways and concurrently nominate CFLAR as potential therapeutic target in these poor-prognosis leukemias.

## Abstract (web summary)

Cut-like homeobox 1 (*CUX1*) is a haploinsufficient tumor suppressor commonly inactivated in acute myeloid leukemia and high-risk myelodysplasia. Here, in a genetically modified murine model, the authors show that *CUX1* deficiency impairs apoptosis leading to leukemia when combined with mutant Flt3-ITD.

## Introduction

Cancers arise following the accumulation of somatic genetic lesions which can activate oncogenes or disable tumor suppressors. While biallelic inactivation of tumor suppressor genes is a well-established mechanism underpinning tumorigenesis^1^, the contribution of tumor suppressor gene haploinsufficiency in tumor development is less well characterized. In this scenario, monoallelic loss of the tumor suppressor is sufficient to promote tumor development. The human genome harbors around 1,400 putative transcription factor genes^2^ of which ~100 genes have been found to be mutated in cancer and approximately half of these genes are implicated as tumor suppressors^3^, with only a handful of these genes implicated as haploinsufficient tumor suppressors^4^. Although some progress has been made in the therapeutic targeting of gain-of-function oncogenic transcription factors^5^, loss-of-function transcription factor tumor suppressors represent difficult targets for direct therapeutic intervention, necessitating alternative approaches to target malignancies harboring such lesions. It is recognized that cancer cells can develop critical dependencies on cellular pathways that abrogate cancer-induced cellular stresses—a phenomenon known as non-oncogene addiction^6,7^. For instance, *KRAS*, *BRAF* and *MYC* oncogenes can induce proteotoxic, DNA replication, mitotic, metabolic and oxidative stresses, that must be countered within cancer cells for their survival^8–14^. While targeting these buffering pathways represents an attractive, alternative strategy to treat cancers driven by oncogenes, it remains unclear whether tumor suppressor genes—and in particular haploinsufficiency of these genes—can engender cells with similar vulnerabilities that can be exploited therapeutically. Moreover, the identification of these actionable cellular dependencies and the molecular mechanisms underlying them remains challenging.

Here, we focussed on the chromosome 7q homeodomain transcription factor *CUX1* (also known as *CUTL1* or *CDP*) which is targeted by monoallelic chromosome 7 deletions [-7/del(7q)] and somatic, heterozygous, truncating mutations in diverse myeloid malignancies such as myelodysplasia (MDS) and myelodysplasia/myeloproliferative neoplasms (MDS/MPN)—both of which can progress to AML^15–19^. *CUX1* mutations are more common in myelomonocytic diseases such as chronic myelomonocytic leukemia (CMML; an MDS/MPN characterized by monocytosis and hematopoietic dysplasia)^18,20^ and high-risk MDS^19^, with biallelic *CUX1* mutations being infrequent, consistent with *CUX1* being predominantly a haploinsufficient tumor suppressor^18,21^. CUX1 harbors four DNA-binding motifs, including three CUT repeats and a homeodomain. While full-length CUX1 (CUX1^p200^) acts as a transcriptional repressor, proteolytic cleavage generates a C-terminal CUX1^p110^ isoform with transcriptionally activating or repressive properties depending on promoter context^22^. Our previous work in mice identified *Cux1* as a potential T-cell leukemia tumor suppressor gene, with *Cux1*-deficient tumors exhibiting increased PI3K signaling, due to loss of CUX1-regulated expression of the gene encoding phosphoinositide 3-kinase interacting protein 1 (PIK3IP1)—a PI3K inhibitor^18,23^. However, the role of *CUX1* loss and *PIK3IP1* in myeloid malignancies remains obscure. Knockdown of *Cux1* in mice to levels approximating *Cux1* haploinsufficiency led to an MDS-like disease, but AML was not apparent, suggesting the need for cooperating genetic lesions^24^. In this regard, genomic studies of *CUX1*-mutated and -7/del(7q) myeloid malignancies have uncovered cooccurring inactivating mutations in other myeloid malignancy-associated genes such as *TET2* and *ASXL1* in addition to gain-of-function mutations in *FLT3* and the *RAS* pathway, but their causal role in *CUX1*-deficient AML development is unclear^19,25–27^. Despite the dismal prognosis conferred by both -7/(del7q) lesions and heterozygous, inactivating *CUX1* mutations in myeloid malignancies^17–19^, which has led to the incorporation of -7/del(7q) lesions in international clinical prognostic scoring systems^15,16^, there are no specific therapies for the treatment of *CUX1*-deficient myeloid malignancies.

Apoptosis evasion is acknowledged to be critical for the development and sustained growth of tumors and is divided into extrinsic and intrinsic (mitochondrial) pathways^28,29^. *CFLAR* (also known as *cFLIP*), encodes three main protein isoforms (c-CFLIP_L_, c-FLIP_S_ and c-FLIP_R_) which can act as antagonists of death receptor-mediated extrinsic apoptosis by competing with caspase-8 during recruitment to ligand-activated receptors of the TNF family, thereby curtailing caspase-8 processing and activation^30^. In addition, CFLAR isoforms can modulate cell death via necroptosis: a programmed form of necrosis mediated by receptor-interacting protein kinase (RIPK) and mixed lineage kinase domain-like pseudokinase (MLKL) proteins. RIPK1 is known to phosphorylate and activate RIPK3, which in turn phosphorylates the key necroptosis effector, MLKL, leading to protein oligomerization and translocation to the plasma membrane^31–36^. In this study, we sought to identify genetic vulnerabilities in *CUX1*-deficient AML cells using CRISPR/Cas9 screening to assess whether *CUX1* deficiency invokes adaptive cellular pathways critical for leukemia survival. Using a murine *Cux1*-deficient leukemia model and human cells, we establish that *CUX1*-deficient leukemia cells rely on elevated levels of CFLAR to evade apoptosis. Mechanistically, we show that *CUX1* haploinsufficiency alleviates CUX1-mediated transcriptional repression of *CFLAR* expression to abrogate cellular apoptosis. Our study illuminates the molecular pathogenesis of *CUX1*-deficient leukemias and establishes that tumor suppressor gene haploinsufficiency can provoke collateral addiction to adaptive cellular pathways of potential therapeutic importance.

## Results

### CRISPR/Cas9 screening identifies *CFLAR* as a selective vulnerability in *CUX1*-deficient cells

**Fig. 1:**
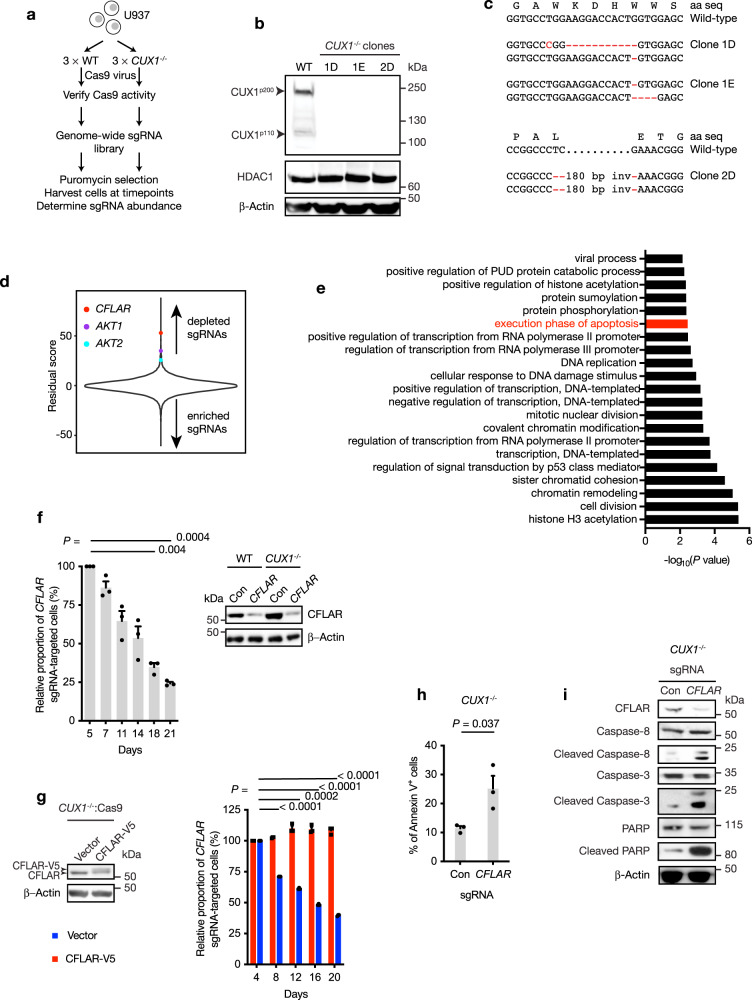
CRISPR/Cas9 drop-out screening identifies genetic vulnerabilities in *CUX1*-deficient cells. **a** Scheme of CRISPR/Cas9 drop-out screen to identify selective vulnerabilities in *CUX1*^*−/−*^ cells. WT, wild-type. **b** Immunoblot showing loss of CUX1 protein expression in three *CUX1*^*−/−*^ clones compared with wild-type (WT). HDAC1 and β-Actin were used as loading controls. The experiment was done twice with similar results. **c** Partial sequence of *CUX1* exon 18 from *CUX1*^*−/−*^ clones 1D, 1E and 2D showing wild-type genomic and amino acid (aa) sequences (top) and clone-specific mutated sequences (bottom); inv, inversion. **d** Distribution of residual scores as a measure of gene essentiality in *CUX1*^*−/−*^ cells compared with wild-type cells. Positions of *CFLAR*, *AKT1* and *AKT2* genes are highlighted. Target genes with higher residual scores exhibit higher degree of potential synthetic lethality in *CUX1*^*−/−*^ than in wild-type cells. **e** Functional annotation of dependency genes in *CUX1*^*−/−*^ cells identified by CRISPR/Cas9 screening. PUD, proteasomal ubiquitin-dependent. *P* values were not adjusted for multiple comparisons. Apoptosis pathway is highlighted in red. **f** Competitive sgRNA assay (left) showing preferential loss of *CFLAR* sgRNA-targeted cells in *CUX1*^*−/−*^ U937 cells compared with wild-type. The results from *CUX1*^*−/−*^ U937 cells were normalized to wild-type cells at each time point. Day 5 was designated a starting value of 100% and subsequent values were calculated relative to day 5. Immunoblot (right) confirming depletion of target protein by *CFLAR*-targeting sgRNA compared with control sgRNA (Con) in wild-type and *CUX1*^*−/−*^ cells. The experiment was done three times. **g** Immunoblot showing expression of CFLAR-V5 in *CUX1*^*−/−*^:Cas9 cells (left) and rescue of lethality following *CFLAR*-targeting sgRNA infection in CFLAR-V5-expressing cells (right). Empty vector was used as control (vector). Results were normalized to day 4 in each case. Statistically significant comparisons shown are between empty vector-expressing groups. The experiment was done three times. **h** Percentage of Annexin-V^+^ cells at day 5 following *CFLAR*-targeting sgRNA infection compared with control sgRNA (Con) in *CUX1*^*−/−*^ cells. The experiment was done three times. **i** Immunoblot of indicated proteins at day 5 following *CFLAR*-targeting sgRNA infection compared with control sgRNA (Con) in *CUX1*^*−/−*^ cells. The experiment was done twice with similar results. *CUX1*^*−/−*^ clone 1D cell line was used in **f**–**i**. One-way ANOVA with Dunnett’s test for multiple comparisons and repeated measures (**f**, **g**), Two-tailed, unpaired *t*-test (**h**).

In an initial approach to determine whether tumor suppressor loss could induce adaptive cellular responses vital for survival, we performed genome-wide CRISPR/Cas9 drop-out screens in isogenic myeloid cancer cells that differed solely in *CUX1* status (Fig. 1a). We selected U937 cells since they have a myelomonocytic phenotype in addition to being deficient in *TET2*^37^; these characteristics are enriched in *CUX1*-mutated myeloid malignancies^18,19^. Furthermore, they represent one of the few myelomonocytic AML lines without pre-existing chromosome 7 copy number alterations, rendering them a feasible background for our approach. Three clonal *CUX1*^*−/−*^ and three *CUX1*-wild-type U937 cell lines were generated by transiently transfecting cells with plasmids engineered to express single guide RNAs (sgRNAs) targeting *CUX1* exon 18, Cas9 endonuclease and EGFP. Single EGFP^+^ clones were expanded in culture for genotyping and immunoblotting to confirm *CUX1* disruption (Fig. 1b, c). Loss of CUX1 expression did not affect proliferation, cell cycle distribution, immunophenotype or steady-state apoptosis of the cells in vitro compared with wild-type cells (Supplementary Fig. 1a–d).

Each cell line was infected with lentiviral supernatants to express comparable functional levels of Cas9 endonuclease (Supplementary Fig. 1e, f). CRISPR/Cas9 drop-out screening was performed using an sgRNA library containing 90,709 sgRNAs targeting 18,010 genes at 200-fold coverage (see Methods). Whereas sgRNAs targeting known essential genes were depleted from both experimental groups as expected, the abundance of the majority of sgRNAs, including control sgRNAs, were not preferentially altered in *CUX1*^*−/−*^ cells compared with wild-type after 21 days from sgRNA library infection (Fig. 1d and Supplementary Data 1). Remarkably, using a Z score threshold > 3.5, we identified 98 selective dependency genes in *CUX1*^*−/−*^ cells including both *AKT1* and *AKT2*, with respective ranks at positions 24 and 96 in our screen (Z score; *AKT1* = 4.81, *AKT2* = 3.53), suggesting that *CUX1*-deficient AML cells could also be more dependent on PI3K signaling consistent with our previously reported sensitivity of *CUX1*-deficient T-cell leukemias to PI3K inhibition^18^ (Fig. 1d).

Gene ontology analysis implicated a number of additional cellular processes which were important for cell survival in *CUX1*-deficient cells, including genes involved in apoptosis regulation (Fig. 1e and Supplementary Data 2). Notably, previous work has shown that *Fas*-deficient mice display expanded myeloid progenitor numbers and concurrent expression of BCL2 in a *Fas*-deficient background can precipitate AML in double-mutant mice^38^, implying that defective apoptosis control could be important in AML pathogenesis. Among the top-ranked genes, *CFLAR* was the most highly positioned apoptosis-regulating gene in our screen (fifth ranked, Z score = 7.28; Fig. 1d), leading us to focus on *CFLAR* as a specific vulnerability in *CUX1*-deficient cells. Interestingly and in contrast, sgRNAs targeting pro-apoptotic genes such as *BCL2L11* (also known as *BIM*), *BCL2L15* and *BCL7B* were enriched in the *CUX1*-deficient cells, suggesting that apoptosis attenuation promoted survival of *CUX1*-deficient cells compared with wild-type. To verify our screening results, we performed competitive sgRNA depletion assays using individual *CFLAR*-targeting or control sgRNAs linked to BFP in two *CUX1*^*−/−*^ and wild-type cell line clones. Expression of *CFLAR*-targeting sgRNA in *CUX1*^*−/−*^ or wild-type cells led to greater depletion of targeted cells in *CUX1*^*−/−*^ compared with wild-type backgrounds, while expression of control sgRNA had no significant effect (Fig. 1f and Supplementary Fig. 1g–i). We noted that cells remaining at 21 days following infection with *CFLAR*-targeting sgRNAs escaped *CFLAR* depletion, which likely accounts for their survival at this time (Supplementary Fig. 1j). Additionally, ectopic expression of a CRISPR/Cas9 cleavage-resistant *CFLAR* isoform in *CUX1*^*−/−*^ cells, abolished the negative impact of *CFLAR*-targeting sgRNA expression on proliferation, excluding off-target effects from the *CFLAR*-targeting sgRNA as the cause of our observed phenotype (Fig. 1g).

We next sought to investigate the basis of *CFLAR* dependency in *CUX1*-deficient cells. We noted that only CFLAR isoforms corresponding to the long c-CFLIP_L_ protein variant were detected in U937 cells. Since this CFLAR isoform has been ascribed both anti- and pro-apoptotic functions^39^, we evaluated the impact of *CFLAR* depletion in *CUX1*^*−/−*^ cells. We found that *CFLAR* depletion led to an increase in Annexin-V^+^ apoptotic cells (Fig. 1h) and cleaved forms of caspase-3, caspase-8 and PARP indicative of activation of the apoptosis-signaling cascade (Fig. 1i), suggesting that *CFLAR* acts to restrain apoptosis. To exclude the possibility that *CFLAR* dependency was unique to *CUX1*^*−/−*^ U937 cells, we reproduced the selective impact of *CFLAR* depletion in engineered *CUX1*^*−/−*^ and wild-type THP-1 myeloid cancer cells, which also possess two wild-type *CUX1* alleles (Supplementary Fig. 1k–m). Furthermore, since *CUX1* haploinsufficiency predominates over complete *CUX1* loss in myeloid malignancies, we wished to establish the relevance of findings by comparing the impact of *CFLAR* depletion in *CUX1*-haploinsufficient cells. To model *CUX1* haploinsufficiency in U937 and THP-1 cells, we used an shRNA (shCUX1) approach to stably deplete CUX1 to approximately 50% of wild-type levels by selecting for puromycin-resistant cells (Supplementary Fig. 1n). Non-targeting shRNAs (shCon) were used as controls. These cells were transduced with lentiviral supernatants to express Cas9 endonuclease followed by *CFLAR*-targeting sgRNAs linked to BFP. Over time, cells harboring *CFLAR*-targeting sgRNAs as marked by BFP were preferentially depleted in shCUX1 cells compared with shCon cells, supporting the idea that *CFLAR* is a specific vulnerability in both *CUX1*-haploinsufficent and *CUX1*-knockout cells (Supplementary Fig. 1o, p).

### *Cux1* haploinsufficiency does not lead to leukemia

In order to investigate whether *CUX1*-deficient primary leukemic cells are more reliant on *CFLAR*, we sought to generate a *CUX1*-deficient leukemia model. To this end, we generated conditional *Cux1* knockout (*Cux1*^*+/fl*^) mice using CRISPR/Cas9 techniques (Supplementary Fig. 2a–c). *Cux1*^*+/fl*^ mice were crossed to the *Vav-iCre* recombinase strain, in which Cre recombinase expression is driven by the *Vav1* promoter that is active in hematopoietic cells. Efficient *Cux1* deletion in *Vav-iCre*^+^ animals was confirmed by PCR (Supplementary Fig. 2d) and CUX1 protein expression was lost in a *Cux1*^*fl*^ allele-dependent manner without detectable truncated N-terminal CUX1 isoforms (Supplementary Fig. 2e).

At six and twelve months, homozygous *Cux1* deletion in *Cux1*^*fl/fl*^;*Vav-iCre*^*+*^ mice led to progressive macrocytic anemia and leukocytosis due to monocytosis and neutrophilia, which was similarly observed in *Cux1*-knockdown mice^24^ (Supplementary Fig. 3a–e). While a subset of aged *Cux1*^*fl/fl*^;*Vav-iCre*^*+*^ mice eventually developed MDS/MPN resembling human CMML starting around one year of age, characterized by variable splenomegaly, hematopoietic dysplasia and organ infiltration with myelomonocytic cells (Supplementary Fig. 3f–h), no cases of acute leukemia were observed. In contrast, haploinsufficient *Cux1*^*+/fl*^;*Vav-iCre*^*+*^ (referred to as *Cux1*^*+/−*^*)* mice displayed mild macrocytic anemia without significant abnormalities in absolute leukocyte numbers (Supplementary Fig. 3k–m), although myelodysplastic features were evident in blood and bone marrow cytospins (Supplementary Fig. 3f–j). These observations suggested that additional mutations were required for the development of myeloid malignancies such as MDS/MPN and AML that are also associated with *CUX1* haploinsufficiency in humans. Interestingly, platelet counts were elevated in *Cux1*^*fl/fl*^;*Vav-iCre*^*+*^ (*P* = 0.01, Student’s *t*-test) and to a lesser extent in *Cux1*^*+/−*^ (*P* = 0.08, Student’s *t*-test) mice at 12 months of age (Supplementary Fig. 3n), which was associated with increased numbers of megakaryocytes in bone marrow sections compared with control mice, suggesting a role for *Cux1* in megakaryopoiesis (Supplementary Fig. 3o).

### *Cux1* haploinsufficiency cooperates with *Flt3*^*ITD*^ mutation leading to CMML and AML in vivo

**Fig. 2:**
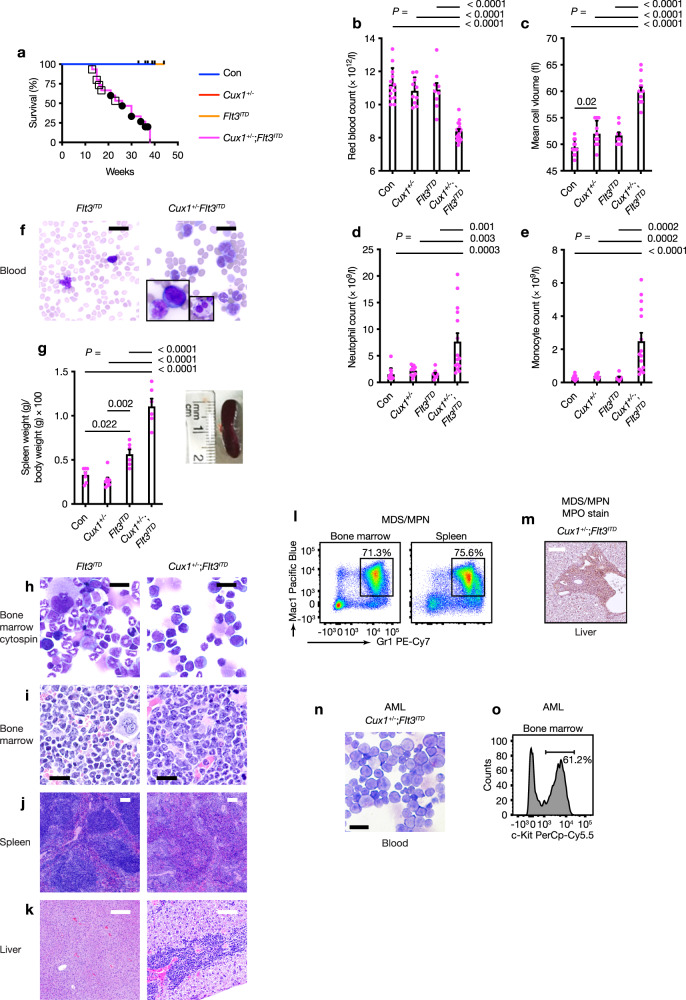
*Flt3*^*ITD*^ mutation cooperates with *Cux1* haploinsufficiency to induce MDS/MPN and AML in vivo. **a** Kaplan–Meier survival curve of *Cux1*^*+/−*^;*Flt3*^*ITD*^ (*n* = 14, magenta line), *Flt3*^*ITD*^ (*n* = 9, orange line), *Cux1*^*+/fl*^*;Vav-iCre*^*+*^ (*Cux1*^*+/−*^) (*n* = 11, red line) and control (*Vav-iCre*^*+/−*^ or Cre-negative littermates, Con, *n* = 11, blue line) mice. Increased mortality in *Cux1*^*+/−*^;*Flt3*^*ITD*^ mice (median survival 28 weeks; control Con versus *Cux1*^*+/−*^;*Flt3*^*ITD*^, *P* < 0.0001; *Cux1*^*+/−*^ versus *Cux1*^*+/−*^;*Flt3*^*ITD*^, *P* < 0.0001; *Flt3*^*ITD*^ versus *Cux1*^*+/−*^;*Flt3*^*ITD*^, *P* = 0.0002; log-rank test). Open square symbols indicate those mice diagnosed with MDS/MPN and black circles indicate AML cases. **b**–**e** Red blood cell count (**b**), red blood cell mean cell volume (**c**) neutrophil count (**d**) and monocyte count (**e**) in control Con (*n* = 13), *Cux1*^*+/−*^ (*n* = 10), *Flt3*^*ITD*^ (*n* = 9) and *Cux1*^*+/−*^;*Flt3*^*ITD*^ (*n* = 15) mice at 10 weeks of age. **f** Representative May-Grünwald-Giemsa (MGG)-stained blood smears from 10-week-old *Flt3*^*ITD*^ (left) and *Cux1*^*+/−*^;*Flt3*^*ITD*^ (right) mice. Insets show an abnormal monocyte and blast cell (left); dysplastic neutrophil (right). Scale bar, 20 µm. Five *Flt3*^*ITD*^ and 10 *Cux1*^*+/−*^;*Flt3*^*ITD*^ mice were assessed. **g** Plot of necropsy spleen weights normalized to body weight (left) from control Con (*n* = 7), *Cux1*^*+/−*^ (*n* = 8), *Flt3*^*ITD*^ (*n* = 6) and *Cux1*^*+/−*^;*Flt3*^*ITD*^ (*n* = 6) mice. Photograph of representative spleen from 10-week-old *Cux1*^*+/−*^;*Flt3*^*ITD*^ mouse (right). **h**–**k** Representative MGG-stained bone marrow cytospin (**h**) and hematoxylin and eosin (H&E)-stained bone marrow (**i**), spleen (**j**) and liver (**k**) sections from 17-week-old *Flt3*^*ITD*^ (left) and *Cux1*^*+/−*^;*Flt3*^*ITD*^ (right) mice. Bone marrow and tissues are infiltrated with excess myelomonocytic cells in the *Cux1*^*+/−*^;*Flt3*^*ITD*^ mouse. Scale bars; black = 20 µm, white = 100 µm. Five *Flt3*^*ITD*^ and 10 *Cux1*^*+/−*^;*Flt3*^*ITD*^ mice were assessed. **l** Flow cytometric plot showing distribution of Gr1^+^ and Mac1^+^ cells in bone marrow (left) and spleen (right) from a 15-week-old *Cux1*^*+/−*^;*Flt3*^*ITD*^ mouse diagnosed with CMML. Percentage of Gr1^+^Mac1^+^ cells is shown. **m** Immunohistochemistry of liver section from a 15-week-old *Cux1*^*+/−*^*;Flt3*^*ITD*^ mouse diagnosed with MDS/MPN showing infiltration with myeloperoxidase (MPO)-expressing cells. Scale bar, 100 µm. Three mice were assessed. **n** Representative MGG-stained blood smear showing blast cells from a 30-week-old *Cux1*^*+/−*^;*Flt3*^*ITD*^ mouse diagnosed with AML. Scale bar, 20 µm. Five mice were assessed. **o** Flow cytometric plot showing percentage of c-Kit^+^ AML cells in bone marrow from a 30-week-old *Cux1*^*+/−*^;*Flt3*^*ITD*^ mouse diagnosed with AML. All plots show mean + s.e.m. Each circle represents one mouse. One-way ANOVA with Tukey’s test for multiple comparisons.

Since we wished to establish a *CUX1*-haploinsufficient AML model, we searched for mutations which cooccur with -7/del(7q) lesions in human AML. As activating FMS-like tyrosine kinase 3 (*FLT3*) mutations are common (~30%) in myeloid malignancies with normal karyotype and are found in ~10% cases with -7/del(7q) lesions^26,27,40,41^, we generated *Cux1*^*+/fl*^;*Flt3*^*ITD/+*^;*Vav-iCre*^*+*^ (herein referred to as *Cux1*^*+/−*^;*Flt3*^*ITD*^) mice to model *Cux1* haploinsufficiency in the context of a heterozygous, activating *Flt3*^*ITD*^ mutation—which alone leads to chronic myeloproliferative disease but not AML in knock-in mice^40,42^. Strikingly, all *Cux1*^*+/−*^;*Flt3*^*ITD*^ mice developed lethal hematologic disease with a median survival of 28 weeks, while control (Con) and single-allele (*Cux1*^*+/−*^ and *Flt3*^*ITD*^) groups remained healthy (Fig. 2a). Blood from 10-week-old *Cux1*^*+/−*^;*Flt3*^*ITD*^ mice revealed macrocytic anemia (Fig. 2b, c), neutrophilia and monocytosis (Fig. 2d, e). Blood smears confirmed leukocytosis with dysplastic monocytes and neutrophils (Fig. 2f). In addition, splenomegaly was evident in *Cux1*^*+/−*^;*Flt3*^*ITD*^ mice (Fig. 2g). Surprisingly, while pathologic examination of moribund *Cux1*^*+/−*^*Flt3*^*ITD*^ mice culled at an earlier age revealed infiltration of liver, spleen and bone marrow with excessive numbers of myelomonocytic cells leading to effacement of normal tissue architecture compatible with a diagnosis of CMML (Fig. 2h–k; square symbols Fig. 2a), older animals succumbed to AML following on from prior CMML (circle symbols Fig. 2a). In CMML cases, increased mature myeloid cell numbers were evident by flow cytometry (Fig. 2l) and myeloperoxidase immunohistochemistry (Fig. 2m), while AML blasts were c-Kit^+^ (Fig. 2n, o).

**Fig. 3:**
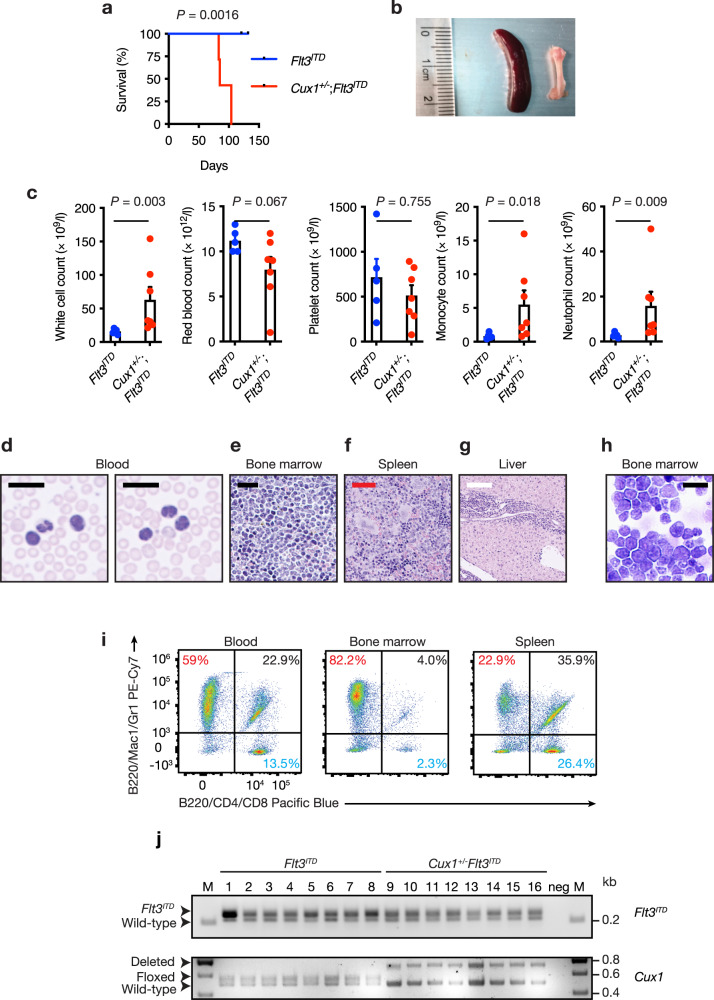
Transplantability of *Cux1*^*+/−*^;*Flt3*^*ITD*^ leukemia. **a** Kaplan–Meier survival curve of irradiated CD45.1^+^ wild-type mice transplanted with 2 × 10^5^ *Cux1*^*+/−*^;*Flt3*^*ITD*^ (*n* = 7) or *Flt3*^*ITD*^ (*n* = 5) bone marrow cells, along with the same number of CD45.1^+^ wild-type support bone marrow cells. Bone marrow cells from two *Cux1*^*+/−*^;*Flt3*^*ITD*^ mice diagnosed with AML at 30 weeks of age were injected into three or four irradiated recipients. *Flt3*^*ITD*^ donor cells were obtained from two animals from a similar age as *Cux1*^*+/−*^;*Flt3*^*ITD*^ mice and injected into two or three recipients. Median survival in *Cux1*^*+/−*^;*Flt3*^*ITD*^ cell recipients was 85 days. *P* = 0.0016, log-rank test. **b** Representative photograph of an enlarged spleen (left) and pale femur (right) from AML-diseased 85-day-old mouse transplanted with *Cux1*^*+/−*^;*Flt3*^*ITD*^ bone marrow cells. **c** Blood count parameters from mice transplanted with cells of the indicated genotypes at 12 weeks post transplantation. Higher white cell counts due to increased monocyte and neutrophil numbers are seen in mice transplanted with *Cux1*^*+/−*^;*Flt3*^*ITD*^ bone marrow cells. **d**–**g** MGG-stained blood smears (**d**) showing abnormal circulating neutrophils and monocytes and H&E-stained bone marrow (**e**), spleen (**f**) and liver (**g**) sections showing myelomonocytic cell tissue infiltration compromising normal tissue architecture. Five mice were assessed. **h** MGG-stained bone marrow cytospin showing AML blasts from an 85-day-old mouse transplanted with *Cux1*^*+/−*^;*Flt3*^*ITD*^ bone marrow cells. Scale bars; black = 20 µm, red = 50 µm, white = 100 µm. Five mice were assessed. **i** Flow cytometric plots showing increased proportion of myeloid (Gr1^+^Mac1^+^, red) cells within blood, bone marrow and spleen. The proportions of B220^+^ B cells (black) and CD4^+^CD8^+^ T cells (blue) are also shown. **j** Agarose gel image showing PCR genotyping for *Flt3*^*ITD*^ (top) and *Cux1* (bottom) alleles in *Flt3*^*ITD*^ (*n* = 8, lanes 1-8) and *Cux1*^*+/−*^;*Flt3*^*ITD*^ (*n* = 8, lanes 9-16) mice. M, DNA ladder; neg, no DNA template. Identity of PCR products is shown on the left. Each circle represents one mouse. Plots show mean + s.e.m. Two-tailed, unpaired Mann–Whitney test.

To assess whether leukemias were transplantable, we injected 2 × 10^5^ bone marrow cells from two *Cux1*^*+/−*^;*Flt3*^*ITD*^ mice diagnosed with AML or *Flt3*^*ITD*^ mice along with the same number of wild-type bone marrow support cells into irradiated recipient wild-type mice. While mice injected with *Flt3*^*ITD*^ cells remained healthy during this study, recipients of *Cux1*^*+/−*^;*Flt3*^*ITD*^ cells developed pallor and cachexia which compromised their survival (Fig. 3a). Splenic enlargement and gross pallor of femurs were observed in recipients of *Cux1*^*+/−*^;*Flt3*^*ITD*^ cells (Fig. 3b). Blood count analysis at necropsy revealed anemia and leukocytosis with the latter attributable to increased neutrophils, monocyte and blasts in mice transplanted with *Cux1*^*+/−*^;*Flt3*^*ITD*^ cells, but not *Flt3*^*ITD*^ cells (Fig. 3c). Blood smears from these animals demonstrated numerous hypolobated, dysplastic neutrophils (Fig. 3d). Histopathology showed that bone marrow, spleen and liver tissues were infiltrated with myelomonocytic and immature blast cells as observed in the peripheral blood (Fig. 3e–g) and bone marrow cytospins demonstrated numerous blast cells along with more mature myelomonocytic cells (Fig. 3h). Flow cytometry of blood, bone marrow and spleen revealed an increase in cells expressing Gr1 and Mac1 consistent with myeloid expansion (Fig. 3i). We also genotyped primary leukemias from *Cux1*^*+/−*^;*Flt3*^*ITD*^ mice since copy-neutral loss of heterozygosity at the *Flt3*^*ITD*^ locus leading to doubling of *Flt3*^*ITD*^ gene dosage has been associated with disease progression in murine and human *FLT3*^*ITD*^-associated AML^43,44^, but we found no evidence of this phenomenon at either *Flt3*^*ITD*^ or *Cux1* loci (Fig. 3j), implying that heterozygosity for both alleles was sufficient to promote leukemogenesis. These data indicate that *Cux1* haploinsufficiency is sufficient to promote transplantable AML in concert with the *Flt3*^*ITD*^ allele.

### *Cux1* haploinsufficiency affects hematopoietic stem and progenitor cell development in *Cux1*^*+/−*^;*Flt3*^*ITD*^ mice

**Fig. 4:**
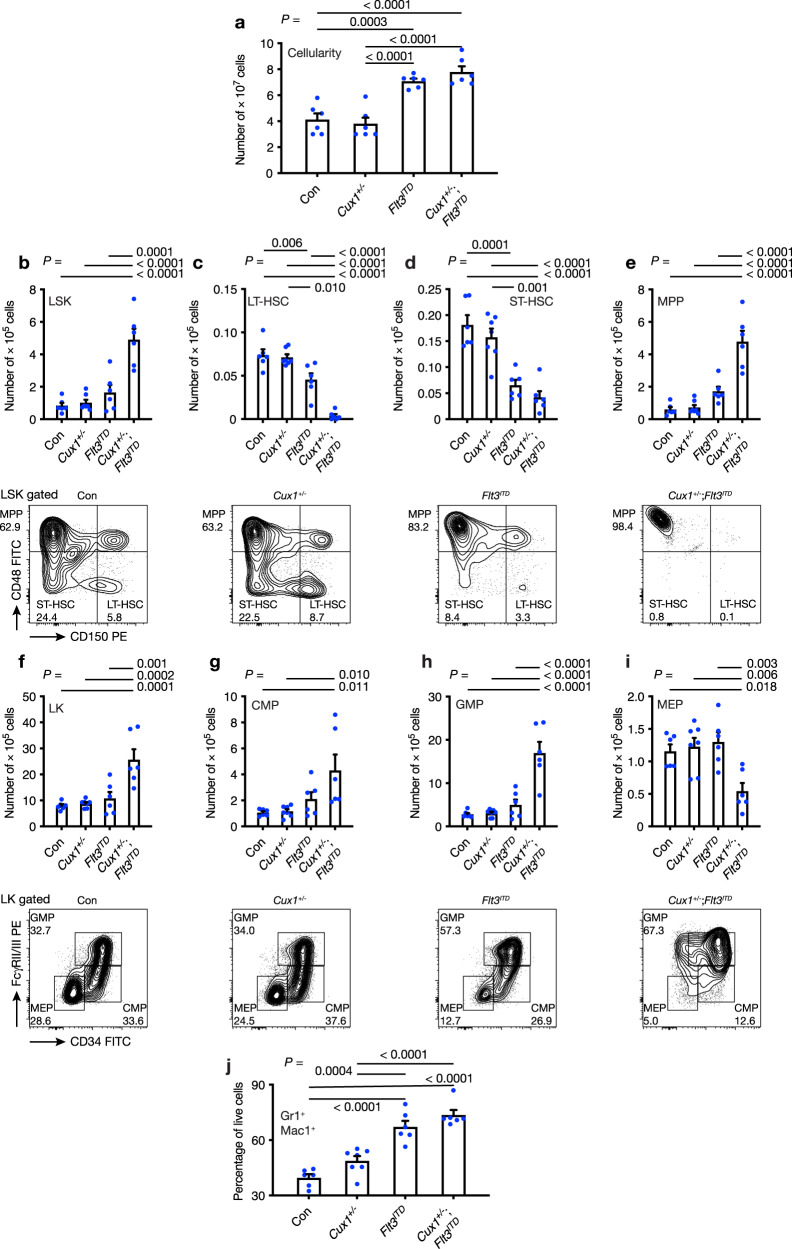
Defective hematopoietic stem and progenitor cell homeostasis in *Cux1*^*+/−*^;*Flt3*^*ITD*^ mice. **a** Total bone marrow cellularity of two legs from control Con (*n* = 6), *Cux1*^*+/−*^ (*n* = 7), *Flt3*^*ITD*^ (*n* = 6) and *Cux1*^*+/−*^;*Flt3*^*ITD*^ (*n* = 6) mice at 10-12 weeks of age. **b**–**e** Plots showing absolute numbers of LSK (**b**), LT-HSC (**c**), ST-HSC (**d**) and MPP (**e**) cells in mice from the indicated genotypes. Representative flow cytometry plots are shown below each bar plot. (**f**–**i**) Plots showing absolute numbers of LK (**f**), CMP (**g**), GMP (**h**) and MEP (**i**) cells in mice from the indicated genotypes. Representative flow cytometry plots are shown below each bar plot. **j** Percentage of Gr1^+^Mac1^+^ myeloid cells in the bone marrow of mice from the indicated genotypes (*n* = 6 per group). All plots show mean + s.e.m. Each circle represents one mouse. One-way ANOVA with Tukey’s test for multiple comparisons.

Since *Flt3*^*ITD*^ expression in mice leads to abnormal hematopoietic stem cell (HSC) and myeloid progenitor (MP) differentiation^40^, we determined the frequency of HSCs, MPs and their subsets using flow cytometry in 10-12-week-old control (Con), *Cux1*^*+/−*^*, Flt3*^*ITD*^ and *Cux1*^*+/−*^*;Flt3*^*ITD*^ mice. Bone marrow cellularity was increased in *Cux1*^*+/−*^*;Flt3*^*ITD*^ and, to a lesser extent, in *Flt3*^*ITD*^ mice (Fig. 4a). Absolute numbers of LSK (Lin^-^Sca-1^+^c-Kit^+^) HSCs were increased in *Cux1*^*+/−*^*;Flt3*^*ITD*^ mice, but not significantly increased in other groups at this age (Fig. 4b). Using CD150 and CD48 signaling lymphocytic activation molecule (SLAM) markers, we characterized the LSK compartment further to define phenotypic long-term HSC (LT-HSC), short-term HSC (ST-HSC) and multipotent progenitor (MPP) cells^45^. Strikingly, the increase in LSK cells in *Cux1*^*+/−*^*;Flt3*^*ITD*^ mice was mainly attributable to expansion of the MPP population at the expense of LT-HSC and ST-HSC populations compared with the control group (Fig. 4c–e). Although LT-HSC frequencies are known to be reduced in *Flt3*^*ITD*^ mice^42^, this reduction was even more pronounced in *Cux1*^*+/−*^*;Flt3*^*ITD*^ mice and unaltered in *Cux1*^*+/−*^ mice compared with controls (Fig. 4c). Assessment of LK (Lin^-^Sca-1^-^c-Kit^+^) MP cells in *Cux1*^*+/−*^*;Flt3*^*ITD*^ mice revealed an expansion of absolute numbers of LK MP cells (Fig. 4f), which was mainly due to increased numbers of granulocyte-monocyte progenitors (GMPs) rather than common myeloid progenitors (CMPs), whereas the frequency of megakaryocyte-erythroid progenitors (MEPs) was decreased compared with other groups (Fig. 4g–i). Bone marrow lineage analysis revealed an increase in the proportion of Gr-1^+^Mac1^+^ myeloid cells in *Flt3*^*ITD*^ and *Cux1*^*+/−*^*;Flt3*^*ITD*^ mice compared with other groups (Fig. 4j). Taken together, these data show that *Cux1* haploinsufficiency and *Flt3*^*ITD*^ cooperate to alter hematopoietic stem and progenitor cell homeostasis to a greater extent than each mutant allele alone. The changes in GMP and MEP numbers provide potential explanations for anemia and dominant myelopoiesis observed in *Cux1*^*+/−*^;*Flt3*^*ITD*^ mice.

### Apoptosis defects in *Cux1*^*+/−*^;*Flt3*^*ITD*^ mice

**Fig. 5:**
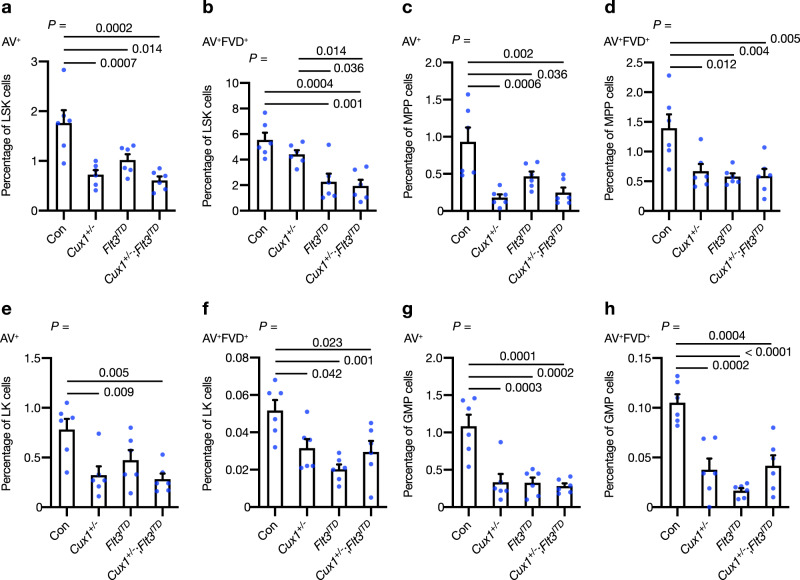
Apoptosis defects in *Cux1*^*+/−*^;*Flt3*^*ITD*^ hematopoietic stem and progenitor cells. **a**, **b** Hematopoietic stem and progenitor cells were identified by flow cytometry and apoptosis within each compartment was determined by staining with Annexin-V (AV) and a fixable viability dye (FVD). Percentage of early- (AV^+^) (**a**) and late- (AV^+^FVD^+^) (**b**) apoptotic cells in LSK compartments of mice from control Con, *Cux1*^*+/−*^, *Flt3*^*ITD*^ and *Cux1*^*+/−*^;*Flt3*^*ITD*^ genotypes at 10-12 weeks of age (*n* = 6 per group). **c**–**h** Percentage of AV^+^ (**c**, **e**, **g**) and AV^+^FVD^+^ (**d**, **f**, **h**) apoptotic cells in MPP (**c**, **d**), LK (**e**, **f**) and GMP (**g**, **h**) compartments of mice from the indicated genotypes at 10-12 weeks of age (*n* = 6 per group). All plots show mean + s.e.m. Each circle represents one mouse. One-way ANOVA with Tukey’s test for multiple comparisons.

Previous analysis of *Flt3*^*ITD*^ mice has shown that *Flt3*^*ITD*^ expression is associated with reduced apoptosis of both LSK HSCs and LK myeloid progenitors in a dose-dependent manner^40^. To assess the apoptosis status of *Cux1*^*+/−*^;*Flt3*^*ITD*^ cells compared with other groups, we performed flow cytometry for Annexin-V (AV) in LSK and LK cell compartments using 10–12-week-old mice. A fixable viability dye (FVD) was used to detect dead cells. *Cux1*^*+/−*^ and *Cux1*^*+/−*^;*Flt3*^*ITD*^ LSK HSCs exhibited a reduced frequency of early apoptotic (AV^+^) cells (Fig. 5a). Concurrent *Cux1* haploinsufficiency and *Flt3*^*ITD*^ mutation was associated with a further reduction in late-apoptotic (AV^+^FVD^+^) cells than observed in single-allele mutant groups (Fig. 5b). We also examined the frequency of apoptotic cells within the SLAM compartment of LSK cells to find reductions in early- and late-apoptotic cells in MPP cells of *Cux1*^*+/−*^, *Flt3*^*ITD*^ and *Cux1*^*+/−*^;*Flt3*^*ITD*^ cells (Fig. 5c, d). While the frequency of apoptotic cells appeared to be lower in *Cux1*^*+/−*^;*Flt3*^*ITD*^ ST-HSCs compared with control, the paucity of these cells (and LT-HSCs) in *Cux1*^*+/−*^;*Flt3*^*ITD*^ mice may have hindered their analysis (Supplementary Fig. 4a–d). Assessment of apoptosis in LK cells revealed reductions in early- and/or late-apoptotic cells in *Cux1*^*+/−*^, *Flt3*^*ITD*^ and *Cux1*^*+/−*^;*Flt3*^*ITD*^ mice compared with control (Fig. 5e, f), which was mainly attributable to decreased early- and late-apoptosis in GMP cells over CMPs and MEPs (Fig. 5g, h and Supplementary Fig. 4e–h). When the higher bone marrow cellularity of *Cux1*^*+/−*^;*Flt3*^*ITD*^ mice was taken into account, reductions in early- and late-apoptotic absolute cell numbers were most marked in LT-HSC and ST-HSC cells of these mice (Supplementary Fig. 4i–t). To explore the status of apoptosis in hematopoietic subsets using an alternative approach, we performed flow cytometry using antibodies against the active form of caspase-3 (C3). Using this approach, we confirmed a reduction in early- (C3^+^) and late- (C3^+^FVD^+^) apoptotic cells in LSK cells from *Cux1*^*+/−*^;*Flt3*^*ITD*^ mice consistent with our Annexin-V data, which we attributed to the MPP subcompartment (Supplementary Fig 4). The frequency of early-apoptotic cells was also lower in LK cells, and in particular GMPs, from *Cux1*^*+/−*^;*Flt3*^*ITD*^ mice, although the changes appeared to be less marked compared with those observed using Annexin-V staining (Supplementary Fig. 4). Collectively, these data demonstrate that apoptosis is perturbed in hematopoietic stem and progenitor compartments of *Cux1*^*+/−*^;*Flt3*^*ITD*^ mice.

Using similar staining approaches, we assessed the apoptosis status within bone marrow lineage compartments. While the frequency of bone marrow B220^+^ B cells was reduced in *Cux1*^*+/−*^;*Flt3*^*ITD*^ mice, probably due to the established effect of *Flt3*^*ITD*^ expression on B-cell development^40^, we did not observe any significant changes in apoptosis in these cells (Supplementary Fig. 5a, c). In contrast, Annexin-V^+^ cells were increased in CD4^+^CD8^+^ T cells of *Cux1*^*+/−*^;*Flt3*^*ITD*^ mice, although the frequency of T cells was similar to control (Supplementary Fig. 5b, d). Intriguingly, apoptosis analysis of Gr1^+^Mac1^+^ myeloid cells was conflicting using Annexin-V and active caspase-3 staining suggesting that apoptosis may not play an important role in the expansion of mature myeloid cells (Supplementary Fig. 5e, h).

To investigate erythropoiesis, we stained bone marrow cells for CD71 and Ter119 antigens to distinguish the distribution of erythroid cells along an established stage I-IV maturation pathway^46^. These markers are able to resolve several erythroid populations: stage I proerythroblasts (CD71^high^;Ter119^med^, stage I); stage II basophilic erythroblasts (CD71^high^;Ter119^high^); stage III polychromatophilic erythroblasts (CD71^med^;Ter119^high^); and stage IV orthochromatophilic erythroblasts (CD71^low^;Ter119^high^). While *Cux1* haploinsufficiency led to a reduction in stage II progenitors, there was no difference in the frequency of Annexin-V^+^ cells compared with control (Supplementary Fig. 5l). Concurrent *Cux1* haploinsufficiency and *Flt3*^*ITD*^ mutation led to reduced frequency of stage II-IV progenitors compared with other groups, consistent with the anemia observed in these mice and alluding a very early defect in erythropoiesis (Supplementary Fig. 5i, j). Intriguingly, the proportion of Annexin-V^+^ cells was reduced in cells from each stage suggesting that non-apoptotic mechanisms are responsible for defective erythropoiesis and anemia (Supplementary Fig. 5k–n).

### Cell cycle progression and transcriptome changes in *Cux1*^*+/−*^;*Flt3*^*ITD*^ cells

Given the reduction in LT-HSCs in *Flt3*^*ITD*^ mice has been partly attributed to loss of stem cell quiescence in this cell population^42^, we assessed the cell cycle status of HSCs and MPs from control, *Cux1*^*+/−*^, *Flt3*^*ITD*^ and *Cux1*^*+/−*^;*Flt3*^*ITD*^ mice using Ki-67 and DAPI staining. More LSK cells from *Cux1*^*+/−*^;*Flt3*^*ITD*^ mice were in G1 and S/G2/M phases of the cell cycle compared with other groups indicating a loss of stem cell quiescence (Supplementary Fig. 6a). Using SLAM markers, we found that cell cycle progression was most pronounced in MPP cells with LT-HSCs being affected to a lesser extent, while ST-HSCs did not demonstrate any abnormalities in the distribution of G0 and G1 cells (Supplementary Fig. 6b–d). Analysis of myeloid progenitors revealed a similar fall in the proportion of G0 cells in *Cux1*^*+/−*^;*Flt3*^*ITD*^ mice with a concomitant increase in G1 cells (Supplementary Fig. 6e) which affected GMPs, CMPs and MEPs (Supplementary Fig. 6f–h). Taken together, these data reveal abnormal loss of hematopoietic stem and progenitor cell quiescence in *Cux1*^*+/−*^;*Flt3*^*ITD*^ mice which could contribute to both the depletion of stem cells and proliferation of more differentiated hematopoietic cell types.

To investigate further the basis of the cooperative leukemic phenotype between *Flt3*^*ITD*^ and *Cux1* haploinsufficiency, we performed transcriptome analysis of LSK cells isolated from *Cux1*^*+/−*^;*Flt3*^*ITD*^ and *Flt3*^*ITD*^ mice. We observed upregulation and downregulation of 297 and 347 genes respectively between *Cux1*^*+/−*^;*Flt3*^*ITD*^ and *Flt3*^*ITD*^ LSK cells (fold change ≥1.5, *P*_adj_ ≤ 0.05; Supplementary Data 3). Gene ontology analysis revealed that *Cux1*^*+/−*^;*Flt3*^*ITD*^ LSK cells exhibited an enrichment in genes involved in apoptosis, intracellular cell signaling, cell proliferation and differentiation compared with *Flt3*^*ITD*^ cells (Supplementary Fig. 7a), while gene set enrichment analysis (GSEA) demonstrated that *Cux1*^*+/−*^;*Flt3*^*ITD*^ LSK cells displayed an enrichment in myeloid cell development and monocyte differentiation signatures (Supplementary Fig. 7b). Collectively, these analyses are in keeping with the increased myelomonocytic lineage output and phenotypes observed in *Cux1*^*+/−*^;*Flt3*^*ITD*^ hematopoietic stem and myeloid progenitor cells.

### CFLAR restrains apoptosis in primary *CUX1*-haploinsufficient hematopoietic cells and represents an in vivo therapeutic target

**Fig. 6:**
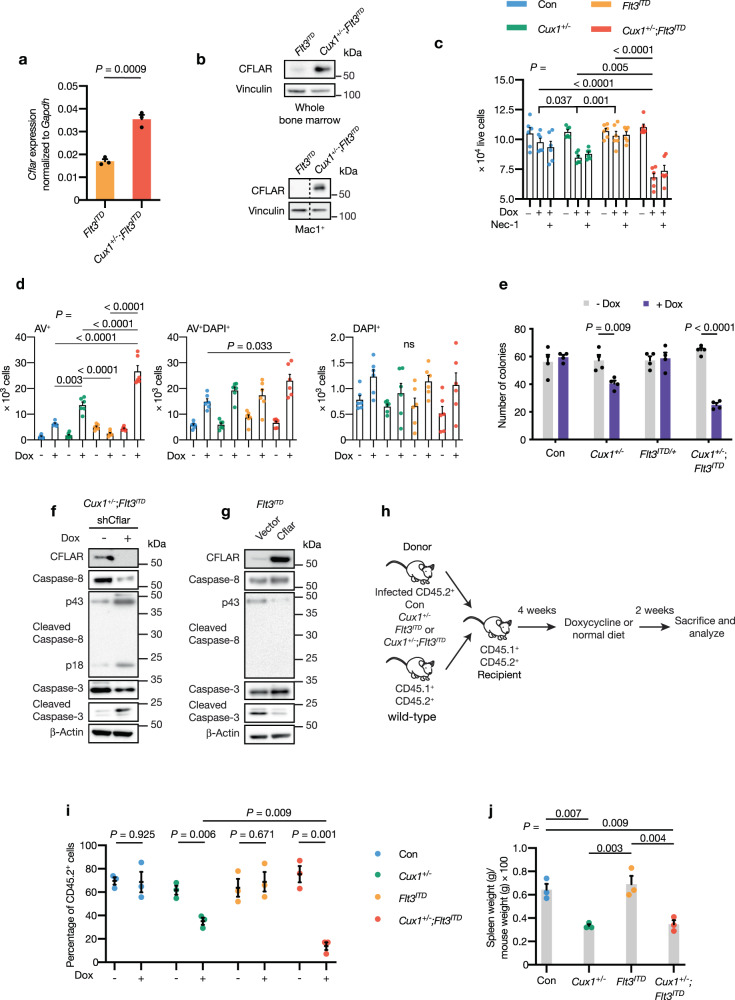
*CFLAR* is a genetic vulnerability and restrains apoptosis in *CUX1*-deficient AML. **a** *Cflar* transcript expression by qRT-PCR in *Cux1*^*+/−*^;*Flt3*^*ITD*^ and *Flt3*^*ITD*^ Mac1^+^ cells. Expression was normalized to *Gapdh*. Biological and technical triplicates were analyzed. **b** Immunoblot showing increased CFLAR expression in whole bone marrow (top) and Mac1^+^ myeloid cells (bottom) from *Flt3*^*ITD*^ and *Cux1*^*+/−*^;*Flt3*^*ITD*^ mice. Vinculin was used as a loading control. The dashed line indicates splicing of non-adjacent lanes from the same blot. The experiment was performed twice. **c** Growth assays using 2 × 10^4^ c-Kit^+^ cells from two mice of the indicated genotypes plated in triplicate. Doxycycline (0.1 µg/ml, Dox) and/or Nec-1 (20 µM) was added and the number of live cells per well was determined by Annexin-V/DAPI staining 48 h later. **d** Annexin-V/DAPI staining to determine numbers of early- and late-apoptotic cells in doxycycline-treated cells from **c**. **e** Quantification of colonies derived from plating 500 c-Kit^+^ cells transduced with doxycycline-inducible *Cflar*-targeting shRNA lentiviruses from indicated mice. Cells from two mice were plated in duplicate on cytokine-supplemented methylcellulose in the presence or absence of doxycycline. Colonies containing more than 50 cells were scored seven days after plating. **f** Immunoblot of lysates from *Cux1*^*+/−*^;*Flt3*^*ITD*^ c-Kit^+^ cells transduced with doxycycline-inducible *Cflar*-targeting shRNA lentiviruses with and without 48 h of doxycycline treatment showing differences in caspase-8 and -3 activation. The immunoblot was performed once. **g** Immunoblot of lysates from *Flt3*^*ITD*^ c-Kit^+^ cells showing effect of stable CFLAR expression on caspase-8 and -3 activation. The immunoblot was performed once. **h** Experimental outline of bone marrow transplant assay to assess selective impact of *Cflar* depletion on *Cux1*^*+/−*^;*Flt3*^*ITD*^ cells compared with control (Con), *Cux1*^*+/−*^ and *Flt3*^*ITD*^ cells. Hematopoietic progenitor cKit^+^ cells from CD45.2^+^ mice of each genotype were infected with doxycycline-inducible *Cflar*-targeting shRNA lentiviruses and 10^5^ puromycin-selected cells were transplanted with 10^5^ wild-type CD45.1^+^CD45.2^+^ bone marrow support cells into lethally irradiated CD45.1^+^CD45.2^+^ recipient mice. After four weeks, mice were fed a doxycycline-containing diet to induce *Cflar* depletion or maintained on a normal diet. Donor-derived CD45.2^+^ bone marrow cells were quantified two weeks later by flow cytometry. **i** Quantification of donor-derived bone marrow CD45.2^+^ cells in recipient mice (*n* = 6, per group) transplanted with control (Con), *Cux1*^*+/−*^, *Flt3*^*ITD*^ or *Cux1*^*+/−*^;*Flt3*^*ITD*^ c-Kit^+^ cells with and without doxycycline-induced *Cflar* depletion. **j** Spleen weights normalized to body weight of mice at the termination of the transplant. All plots show mean + /± s.e.m.; ns, not significant; two-way (**c**) or one-way (**d**, **j**) ANOVA with Tukey’s test for multiple comparisons, Two-tailed, unpaired *t*-test (**a**, **e**, **i**).

Having established that *CFLAR* depletion triggers apoptotic cell death in U937 and THP-1 myeloid cancer cell lines, we sought to test the relevance of our findings in our *Cux1*-haploinsufficient murine leukemia model. We determined CFLAR levels in Mac1^+^ myeloid and whole bone marrow cells from *Cux1*^*+/−*^;*Flt3*^*ITD*^ and *Flt3*^*ITD*^ mice. Consistent with our observations in human myeloid cancer cell lines, *Cflar* transcript and corresponding protein levels were increased in *Cux1*^*+/−*^;*Flt3*^*ITD*^ compared with *Flt3*^*ITD*^ cells (Fig. 6a, b). We next assessed the impact of depleting *Cflar* using shRNA by infecting c-Kit^+^ hematopoietic cells from control (Con), *Cux1*^*+/−*^*, Flt3*^*ITD*^ and *Cux1*^*+/−*^;*Flt3*^*ITD*^ mice with lentiviruses designed to express doxycycline-inducible shRNA targeting *Cflar*. We cultured infected cells in the presence of doxycycline to induce *Cflar* knockdown or drug vehicle as a control. After 48 h, we observed a significant reduction in live cell numbers in *Cux1*^*+/−*^;*Flt3*^*ITD*^ cells compared with other groups following doxycycline treatment (Fig. 6c). A lesser effect was also seen in *Cux1*^*+/−*^ cells (Fig. 6c). Using Annexin-V/DAPI staining, we established that there were greater numbers of early-apoptotic (AV^+^) cells in doxycycline-treated *Cux1*^*+/−*^;*Flt3*^*ITD*^ and *Cux1*^*+/−*^ cells (Fig. 6d). Moreover, late-apoptotic cells (AV^+^DAPI^+^) were increased in *Cux1*^*+/−*^;*Flt3*^*ITD*^ cells compared with control (Fig. 6d). The difference in cell numbers was also apparent in methylcellulose assays, where we observed fewer colonies arising from doxycycline-treated *Cux1*^*+/−*^;*Flt3*^*ITD*^ and *Cux1*^*+/−*^ cells compared with vehicle-treated cells after seven days (Fig. 6e). Importantly, there was no significant difference in colony number from control or *Flt3*^*ITD*^ cells treated in the same way, arguing that *Cflar* is a specific vulnerability in the context of *Cux1* haploinsufficiency (Fig. 6e). To investigate the contribution of apoptosis further, we examined the status of the initiator and executioner caspases downstream of the canonical CFLAR apoptosis pathway following *Cflar* depletion in *Cux1*^*+/−*^;*Flt3*^*ITD*^ c-Kit^+^ cells. Immunoblotting demonstrated that *Cflar* depletion was associated with activation and cleavage of caspase-3 and caspase-8 proteins, consistent with the established role of CFLAR in inhibiting the initiator caspase, caspase-8 (Fig. 6f). In contrast, retroviral restoration of CFLAR in *Flt3*^*ITD*^ c-Kit^+^ cells had the opposite effect on caspase activation (Fig. 6g).

Since CFLAR can also influence cell death through necroptosis, we assessed the contribution of necroptosis to cell death in our model. To this end, we treated c-Kit^+^ cells from each genotype group with a combination of doxycycline and the RIPK1 inhibitor, Necrostatin-1 (Nec-1). We found that Nec-1 treatment was unable to rescue cell viability in doxycycline-treated cells, arguing that necroptosis does not play a significant role in cell death following *Cflar* depletion (Fig. 6c). Furthermore, we did not observe any evidence of MLKL phosphorylation in doxycycline-treated c-Kit^+^ cells from any genotype, again suggesting apoptosis rather than necroptosis is the predominant means of cell death following *Cflar* depletion in our model (Supplementary Fig. 8).

To assess the impact of *Cflar* reduction on leukemia development in vivo, we performed transplant experiments using CD45.2^+^ control (Con), *Cux1*^*+/−*^, *Flt3*^*ITD*^ or *Cux1*^*+/−*^;*Flt3*^*ITD*^ c-Kit^+^ cells transduced with doxycycline-inducible, *Cflar*-targeting shRNA lentiviruses along with wild-type CD45.1^+^CD45.2^+^ support bone marrow cells into lethally irradiated CD45.1^+^CD45.2^+^ recipient mice (Fig. 6h). Four weeks following transplant, we administered doxycycline chow for two weeks to experimental groups, while control groups were maintained on a normal diet. Flow cytometric analysis revealed that the frequency of transplanted, bone marrow CD45.2^+^ cells was significantly reduced in mice injected with *Cux1*^*+/−*^ or *Cux1*^*+/−*^;*Flt3*^*ITD*^ cells only following doxycycline chow administration compared with mice injected with control or *Flt3*^*ITD*^ cells (Fig. 6i). Furthermore, spleen weights were significantly reduced in doxycycline-treated mice injected with *Cux1*^*+/−*^ or *Cux1*^*+/−*^;*Flt3*^*ITD*^ cells transduced with *Cflar*-targeting shRNAs compared with control and *Flt3*^*ITD*^ groups (Fig. 6j). Collectively, our data support the hypothesis that *Cflar* acts to safeguard against apoptosis in *Cux1*-haploinsufficient cells.

### CUX1 is a direct transcriptional repressor of *CFLAR* expression

**Fig. 7:**
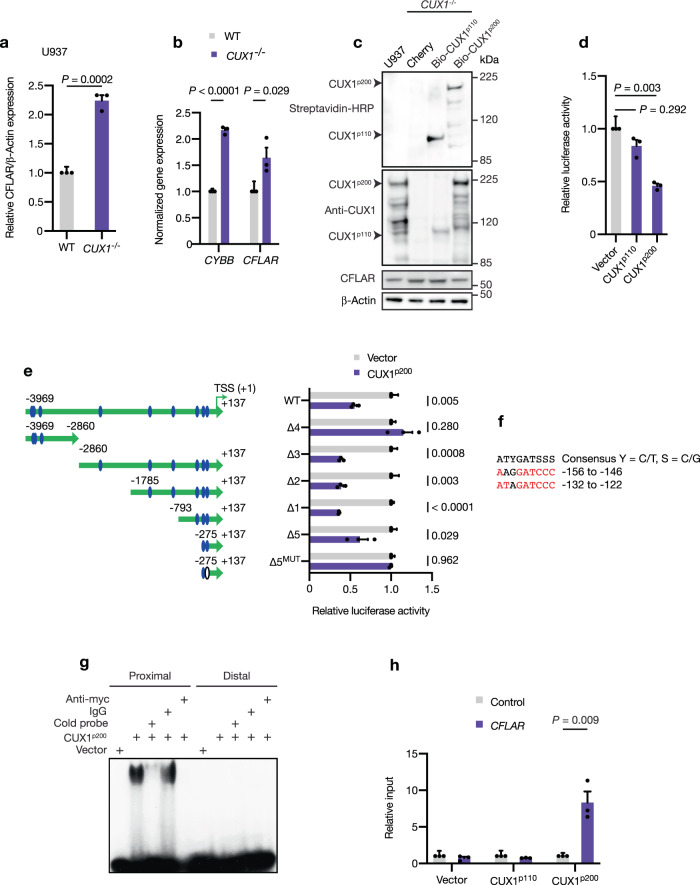
*CFLAR* is directly repressed by CUX1. **a** Quantification of CFLAR protein levels in *CUX1*^*−/−*^ U937 cells compared with wild-type. β-Actin was used as a loading control. The experiment was performed three times. **b** Expression of *CYBB* and *CFLAR* transcripts normalized to *HPRT1* in *CUX1*^*−/−*^ U937 cells compared with wild-type by qRT-PCR. Biological and technical triplicates were analyzed. **c** Immunoblot showing expression of biotinylated CUX1^p100^ and CUX1^p200^ proteins (top) in doxycycline-treated *CUX1*^*−/−*^ U937 cells harboring the indicated constructs. The corresponding levels of CFLAR are also shown (bottom). β-Actin was used as a loading control. The experiment was performed three times. **d** Promoter-luciferase assays showing luciferase activity in HEK293T cells transfected with the indicated constructs and a ~4 kb *CFLAR* promoter-luciferase reporter. Values were normalized to empty vector. Three experiments were performed with triplicate samples in each experiment. **e** Promoter-luciferase assays with deletion constructs of the *CFLAR* promoter-luciferase reporter (large green arrow). Blue ovals, potential CUX1-binding sites. White oval, mutated potential CUX1-binding site. TSS, transcriptional start site (nominated + 1 position). Three experiments were performed with triplicate samples in each experiment. **f** Sequence of potential CUX1-binding sites in *CFLAR* promoter closest to TSS. Locations of binding sites relative to TSS are shown. Consensus CUX1-binding site is shown (top). Red, exact consensus match. **g** Representative electrophoretic mobility shift assay with indicated components and proximal (−132 to −122) or distal (−156 to −146) radiolabeled, potential CUX1-binding dsDNA probes. The experiment was performed twice with similar results. **h** Chromatin immunoprecipitation assay followed by qRT-PCR for the *CFLAR* promoter region (purple) or distal control region (gray) in *CUX1*^*−/−*^ U937 cells complemented with the indicated constructs as shown in **c**. Biological and technical duplicates were analyzed. All plots show mean + s.e.m. Two-tailed, unpaired *t*-test.

Since our data supported the idea that *CFLAR* is a mediator of protective, anti-apoptotic responses in *CUX1*-deficient cells, we sought to determine the basis of this phenomenon and noticed that *CUX1*^*−/−*^ U937 cells displayed higher levels of CFLAR protein expression and cytarabine resistance (Fig. 7a and Supplementary Fig. 9a). CFLAR and CUX1 levels were also reciprocally correlated in tested human AML cell lines and in *CUX1*-knockdown Cancer Cell Line Encyclopedia efforts^47^ (Supplementary Fig. 9b, c). Reverse-phase protein array (RPPA) analysis suggested that *CUX1*^*−/−*^ cells exhibited higher pAKT^S473^ and pAKT^T308^ levels consistent with previously reported AKT pathway activation^18^ (Supplementary Fig. 9d and Supplementary Data 4). In contrast, cleaved caspases-8 levels appeared reduced in agreement with the anti-apoptotic function of CFLAR (Supplementary Fig. 9d, e). Higher transcript levels of *CFLAR* and *CYBB* (whose expression is known to be repressed by CUX1; ref. ^48^) were also observed (Fig. 7b), leading us to hypothesize that CUX1 acts as a direct transcriptional repressor of *CFLAR*. Induction of CUX1^p110^ or CUX1^p200^ protein isoforms, which can be biotinylated, to near physiologic levels or Cherry protein control in *CUX1*^*−/−*^ U937 cells led to suppression of CFLAR by CUX1^p200^ but not CUX1^p110^ or Cherry, consistent with CUX1^p200^ acting as a repressor of *CFLAR* expression (Fig. 7c and Supplementary Fig. 9f). Furthermore, promoter-luciferase assays using a ~4 kb genomic fragment upstream of the *CFLAR* transcriptional start site (TSS), which harbors multiple putative CUX1-binding sites similar to the consensus ATYGATSSS (where Y = C/T, S = C/G), showed that CUX1^p200^ was able to repress promoter-luciferase activity (Fig. 7d). Moreover, assays using deletion constructs narrowed the repressive activity of CUX1^p200^ to a 275 bp region upstream of the TSS, which contains two putative CUX1-binding sites (Fig. 7e, f). By using electrophoretic mobility shift assays, we found that CUX1^p200^ was only able to bind the putative CUX1-binding site nearest to the TSS in specific manner as revealed by cold probe and antibody competition assays (Fig. 7g). Mutation of this site within the promoter construct abolished CUX1^p200^-mediated repression of promoter-luciferase activity (Fig. 7e). Chromatin immunoprecipitation assays followed by qRT-PCR for this and distal control genomic regions using doxycycline-inducible CUX1^p110^, CUX1^p200^ and control cell lines showed that CUX1^p200^ binds selectively to -132 to -122 region of the *CFLAR* promoter in vivo (Fig. 7h). In summary, these data establish CUX1^p200^ as a direct repressor of *CFLAR* expression in vivo and imply that *CUX1* deficiency can lead to *CFLAR* upregulation and associated apoptosis attenuation.

### Primary -7/del(7q) AML cells are sensitive to CFLAR-signaling axis inhibition

**Fig. 8:**
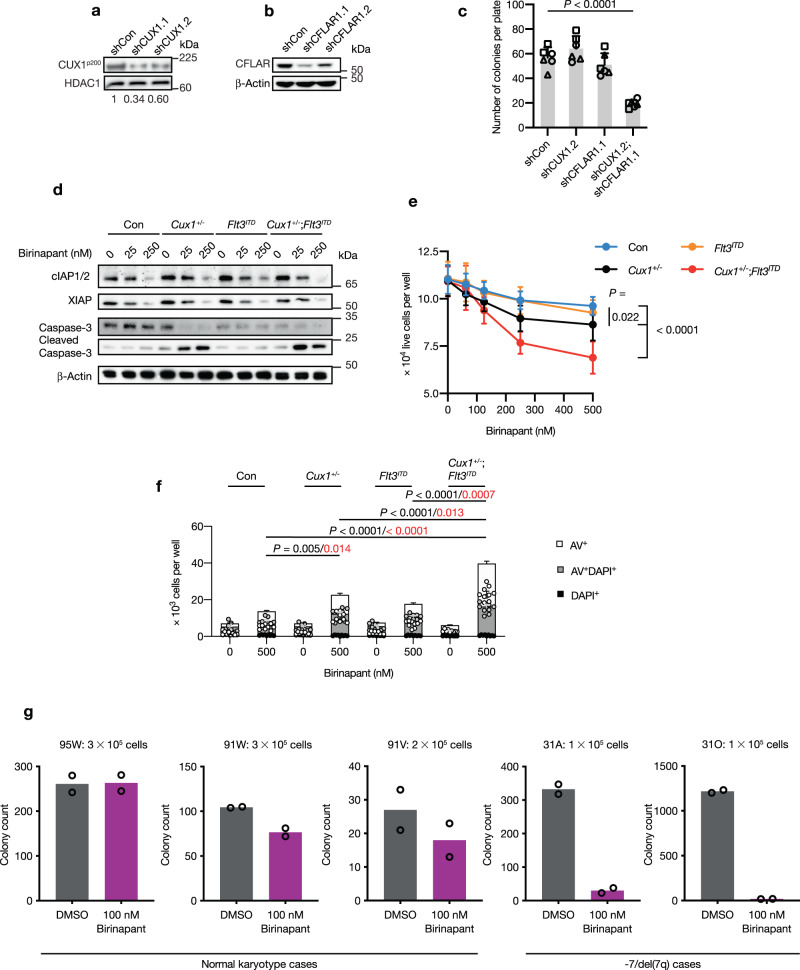
*CUX1*-haploinsufficient cells are sensitive to birinapant treatment. **a**, **b** Immunoblot showing knockdown of *CUX1* (shCUX1.1 and shCUX1.2) (**a**) and *CFLAR* (shCFLAR1.1 and shCFLAR1.2) (**b**) using two independent shRNA vectors. A non-targeting control (shCon) was used as a knockdown control. HDAC1 or β-Actin were used as loading controls. The ratios of CUX1/HDAC1 levels normalized to shCon are shown below the blot in **a**. The blot was performed twice. **c** Methylcellulose colony-forming assays using 500 human CD34^+^ cells transduced with the indicated shRNAs. Each shape represents data from an individual donor (*n* = 3). Cells were plated in duplicate and colonies containing more than 50 cells were scored. **d** Immunoblot showing the changes in the levels of indicated proteins following 48 h of birinapant treatment (0, 25, 250 nM) on control (Con), *Cux1*^*+/−*^, *Flt3*^*ITD*^ or *Cux1*^*+/−*^;*Flt3*^*ITD*^ c-Kit^+^ cells. β-Actin was used as a loading control. The experiment was performed twice. **e** Effect of birinapant treatment (0–500 nM) on growth of 2 × 10^4^ c-Kit^+^ cells from three mice of the indicated genotypes which were plated in triplicate. The number of live cells per well was determined by Annexin-V/DAPI staining 48 h later. **f** Apoptosis assays using Annexin-V/DAPI staining of cells in **e**. The total number of early- and late-apoptotic cells per well for each genotype was determined. Statistical significance was determined for birinapant-treated groups (black, AV^+^ cells; red, AV^+^DAPI^+^ cells). **g** Bar plots of colony counts from methylcellulose colony-forming assays using -7/del(7q) or normal karyotype mononuclear cells from AML patients treated with 100 nM birinapant or DMSO. The number of plated cells is shown above each graph. Cells were plated in duplicate for each patient. Colonies were counted 14 days after plating. Plots show mean ± s.e.m. One-way ANOVA with Dunnett’s test for multiple comparisons (**c**), two-way ANOVA with Tukey’s test for multiple comparisons (**e**), one-way ANOVA with Tukey’s test for multiple comparisons (**f**).

To determine whether *CUX1* deficiency led to similar *CFLAR* dependency in human hematopoietic cells, we designed lentiviral vectors to co-express *CUX1*-targeting shRNAs and GFP or *CFLAR*-targeting shRNAs along with mCherry. Vectors expressing non-targeting shRNAs linked to GFP or mCherry were generated as controls. We assessed the knockdown efficiency of a pair of shRNAs targeting *CUX1* or *CFLAR* in U937 cells and selected shCUX1.2 that resulted in downregulated protein levels close to 50% compared with controls for further study (Fig. 8a, b). Human CD34^+^ cells from healthy donors were transduced with pairs of GFP- and mCherry-expressing shRNA vectors to target *CUX1*, *CFLAR* or non-targeting controls in order to achieve depletion of *CUX1* and/or *CFLAR*. GFP^+^mCherry^+^ double-positive cells were isolated and their colony-forming potential was assessed in methylcellulose assays. While *CUX1* or *CFLAR* depletion alone had no significant impact on colony numbers, combined *CUX1* and *CFLAR* knockdown resulted in significantly fewer colonies compared with non-targeting control, consistent with our findings in our other models (Fig. 8c).

At present no pharmacological inhibitors of CFLAR are routinely available, but second mitochondria-derived activator of caspase (SMAC)-mimetics, such as birinapant, have attracted attention as therapies in cancer, including myeloid malignancies. SMAC-mimetics antagonize IAP family members including cellular IAP (cIAP) and X-linked IAP (XIAP) proteins, which act proximally and distally of CFLAR to promote caspase activation and apoptosis^49^, suggesting an alternative pharmacological, therapeutic avenue. Notably, *XIAP* was ranked at position 147 in our CRISPR/Cas9 screen (Z score = 3.13), suggestive of preferentialvulnerability of *CUX1*-deficient cells to *XIAP* loss. Treatment of c-Kit^+^ cells from control, *Cux1*^*+/−*^, *Flt3*^*ITD*^ and *Cux1*^*+/−*^;*Flt3*^*ITD*^ mice led to dose-dependent reduction in cIAP1/2 and XIAP levels as anticipated (Fig. 8d). We cultured c-Kit^+^ cells from each genotype in the presence of various birinapant concentrations and determined the numbers of live cells after 48 h of treatment. There was a significant birinapant dose-dependent reduction in the number of live cells in *Cux1*^*+/−*^ and *Cux1*^*+/−*^;*Flt3*^*ITD*^ groups compared with the control (Fig. 8e). Furthermore, apoptosis assays revealed an increase in early- and late- apoptotic cells in these two groups following birinapant treatment (Fig. 8f). Consistent with this observation, cleaved caspase-3 levels were increased in these cells by immunoblotting (Fig. 8d).

Given cIAP proteins and their antagonism can potentially affect cell survival by other mechanisms such as necroptosis and NFκB signaling^49^, we assessed the contribution of these pathways to birinapant-induced cell death. The addition of Nec-1 to birinapant-treated cells was unable to ameliorate the loss of viability and birinapant treatment was not associated with MLKL phosphorylation in any genotype group (Supplementary Fig. 10a). Immunoblotting of birinapant-treated cell lysates revealed reduction in NFκB p65 and increased IκBα levels, consistent with suppression of canonical NFκB signaling. Conversely, non-canonical NFκB signaling appeared to be activated as suggested by increased NFκB p52 levels. However, there were no major differences between genotypes (Supplementary Fig. 10b). Taken together, our data show that *Cux1*-haploinsufficient cells exhibit increased sensitivity to birinapant treatment, which is associated with augmented apoptosis.

Finally, to explore the relevance of our findings in primary human leukemia samples, we performed colony-forming assays using mononuclear cells isolated from AML patients with and without -7/del(7q) chromosomal lesions (Supplementary Data 5) treated with birinapant or drug vehicle. Although samples showed genetic heterogeneity and variable colony-forming capacity, the two available -7/del(7q) AML cases displayed sensitivity to birinapant treatment, while normal karyotype and healthy-donor CD34^+^ samples remained largely unaffected, supporting a potential relationship between *CUX1* gene dosage and sensitivity to birinapant (Fig. 8g and Supplementary Fig. 10c).

## Discussion

It is increasingly acknowledged that oncogenes can induce non-oncogene addictions of potential therapeutic importance. Our study indicates that tumor suppressor haploinsufficiency is sufficient to induce an apoptosis stress-mitigating pathway which is essential for leukemic cell survival—a state we refer to as haploinsufficiency-induced gene addiction. Moreover, our data directly link a transcription factor (*CUX1*) in mediating this apoptosis-control pathway addiction through transcriptional regulation of *CFLAR* expression. Our study reinforces the importance of apoptosis control in myeloid leukemogenesis and particularly highlights the significance of the extrinsic apoptosis pathway in AML survival associated with *CUX1* haploinsufficiency. Notably, higher *CFLAR* expression has been associated with inferior survival in one AML cohort^50^ and chemotherapy resistance in several tumor types^51–53^. In contrast to pancreatic cancer, where *CUX1* is oncogenic, highly expressed and suppresses TRAIL-induced apoptosis through unclear mechanisms^54^, we propose that *CUX1* haploinsufficiency in myeloid malignancies confers a reliance on CFLAR-regulated anti-apoptotic survival. How *CUX1* behaves as an oncogene in some tumor types while acting as a tumor suppressor in myeloid malignancies remains unclear, but we speculate that differences in CUX1 target gene expression programs between cell types may ultimately either promote or suppress cellular transformation in a tissue-specific fashion. Uncovering crucial CUX1 target genes involved in tumorigenesis in different cell types is an important goal for future studies.

Haploinsufficiency for *CUX1* due to somatic mutations is associated with inferior prognosis in myeloid malignancies similar to the adverse outcome of -7/del(7q) myeloid malignancies which has been recognized for over three decades^55,56^. Our conditional knockout mice establish that *Cux1* haploinsufficiency requires cooperative mutations such as *Flt3*^*ITD*^ to induce the spectrum of -7/del(7q) myeloid malignancies as observed in humans. As AML emerges from an MDS/MPN disease in our model, it will be interesting to determine whether additional genetic lesions are acquired during leukemia development. In the context of *Flt3*^*ITD*^ mutation, *Cux1* haploinsufficiency leads to dysregulation of hematopoietic stem and progenitor cell homeostasis, which is associated with defects in apoptosis and cell cycle quiescence. Although the direct contribution of *Cflar* to these phenotypes requires further investigation, our data show that *CFLAR* safeguards against apoptotic cell death in *CUX1*-haploinsufficient cells.

While direct inhibitors of CFLAR are in preclinical development^57^, the sensitivity of *CUX1*-haploinsufficient cells to birinapant alludes to a potentially actionable way to treat *CUX1*-deficient myeloid malignancies. While birinapant treatment induces apoptosis in *CUX1*-haploinsufficient cells, we acknowledge that other birinapant-mediated effects on processes such as NFκB signaling could also influence survival outcome. Long-term treatment of our *Cux1*-haploinsufficient leukemia model with birinapant will be interesting to determine the durability of responses. A previous study showed that birinapant increased apoptosis in human AML stem cells compared with CD34^+^ cells from normal individuals^58^. Although a phase 2 study of azacitidine with or without birinapant failed to demonstrate a difference in response rate between the two arms in genetically unselected myeloid malignancy patients^59^, we encourage the development of direct CFLAR inhibitors and the further clinical evaluation of SMAC-mimetics in -7/del(7q) myeloid malignancies. Future studies will aim to examine the efficacy of combining PI3K-AKT pathway inhibition with CFLAR pathway-targeting agents in poor-prognosis -7/del(7q) myeloid malignancies. Finally, since *CUX1* is mutated in a wider spectrum of cancers^18^, our findings merit further scrutiny in other *CUX1*-mutated cancer types.

## Methods

### Mouse strains

Conditional *Cux1* knockout mice, harboring *lox*P sites flanking *Cux1* exons 15-18, were generated by injecting C57BL/6 N zygotes with two in vitro-transcribed T7 promoter-primed sgRNAs (12.5 ng/µl) targeting introns 14 (Cux1_int14) and 18 (Cux1_int18), two complementary *lox*P site-containing donor single-stranded oligonucleotides (Cux1_loxP14 and Cux1_loxP18; 100 ng/µl), along with in vitro-transcribed T7 promoter-primed Cas9 mRNA (50 ng/µl). Oligonucleotides used in this study are listed in Supplementary Data 6. Zygotes were cultured until the blastocyst stage before transplantation into pseudo-pregnant females. Correctly targeted founder mice were identified by PCR using primers P1 and P2 for the first *lox*P site and P3 and P4 for the second *lox*P site along with ear punch genomic DNA isolated from offspring of chimeric mice bred to C57BL/6 N wild-type animals. The identity of PCR products was confirmed by sequencing. Mice were maintained on a C57BL/6 background.

To mediate *Cux1* deletion in hematopoietic tissues, *Cux1*^*+/fl*^ mice were bred with *Vav-iCre* mice (008610, The Jackson Laboratory). *Cux1* deletion was confirmed by PCR using primers P1, P2 and P3 along with genomic DNA from splenocytes or peripheral blood leukocytes. Wild-type, floxed and deleted *Cux1* alleles led to PCR products of 502, 542 and 704 bp respectively when genotyping was performed with P1, P2 and P3 primers using the PCR parameters: 95 °C for 15 min; 35 cycles of 95 °C for 30 s, 58 °C for 30 s, 72 °C for 45 s; and 72 °C for 5 min for the final extension step. *Vav-iCre* mice were genotyped by PCR using VavF and VavR primers. *Flt3*^*ITD*^ mice were genotyped using Flt314F and Flt314R primers. The genotypes of the mice were not blinded or randomized due to experimental design. Mice were maintained in 12 h light:12 h dark cycle environment with an ambient temperature of 21 ± 2 °C and humidity of 55 ± 10%. All procedures were performed in accordance with UK Home Office regulations, UK Animals (Scientific Procedures) Act 1996 and with approval from the Animal Welfare Committee at the Wellcome Sanger Institute.

### Human samples

Human leukemia and CD34^+^ cells were obtained with informed consent from individuals recruited onto the University of Cambridge Blood and Stem Cell Biobank UK REC-approved study (07/MRE05/44). Experiments using human cells were conducted with ethical approval from this study and the Wellcome Sanger Institute. Samples were linked anonymized with linkage information held by the study custodian.

### Mouse pathology

Blood was collected from tail veins into ethylenediaminetetraacetic acid (EDTA)-coated tubes. Automated peripheral blood counts were obtained using a Vet abc machine (scil). Blood smears and bone marrow cytospins were stained with May-Grünwald Giemsa solutions (Sigma). Mouse tissues were fixed in formalin and embedded in paraffin. Blocks were cut into 5 μm sections and mounted onto glass slides prior to staining with hematoxylin and eosin. For immunohistochemistry, slides were microwaved in 10 mM citric acid (pH 6.0) to retrieve antigens. Endogenous peroxidases were quenched using hydrogen peroxide and staining was performed using anti-myeloperoxidase antibody (1:100, ab9535, Abcam) diluted in TBST for 1 h at room temperature. Signal detection was accomplished using an HRP/DAB (ABC) Detection IHC kit (Abcam, ab64264). Sections were counterstained with hematoxylin before dehydration and coverslip mounting. Slide images were captured using a Nanozoomer digital slide scanner (Hamamatsu).

### Flow cytometry and antibodies

Bone marrow cells were obtained by flushing femurs and tibiae with FACS buffer (PBS supplemented with 2% FBS). Red cell-depleted single-cell suspensions were prepared by passing cells through a 70 µm cell strainer and treatment with ACK lysing buffer (Lonza). Cells were treated with Fc Block (BD Biosciences) in cases where anti-CD16/32 staining was not performed. Cells were labeled with monoclonal antibodies or streptavidin-linked fluorophores suspended in FACS buffer for 30 min on ice. All flow cytometry reagents were purchased from eBioscience, BioLegend or BD Biosciences. For flow cytometric analysis of specific hematopoietic populations, lineage-positive cells were identified using Gr1 (RB6-8C5), CD11b (M1/70), Ter119 (TER119), B220 (RA3-6B2), CD4 (RM4-5), CD8 (53-6.7) and CD3 (145-2C11) antibodies conjugated to biotin used at 1:100 dilution. A Lineage Cell Depletion Kit (Miltenyi) was used to deplete lineage-positive cells prior to cell sorting. To reveal hematopoietic populations, cells were incubated with cocktails of the following antibodies used at 1:100 dilution: c-Kit (2B8), Sca1 (D7), CD150 (TC15-12F12.2), CD48 (HM48-1), CD34 (RAM34), CD16/32 (93), which were conjugated to PE, PercP-Cy5.5, PE-Cy7 or FITC. Biotin-conjugated antibodies were revealed using streptavidin conjugated to APC, APC-Cy7 or eFluor 450 used at 1:200 dilution. Live cells were identified by DAPI (Thermo) exclusion or Fixable Viability Dye eFluor 780 (eBioscience). Populations were defined as follows: LT-HSCs, CD150^+^CD48^-^LSK; ST-HSC, CD150^-^CD48^-^LSK; MPP, CD150^-^CD48^+^LSK; GMPs, Lin^-^Sca-1^-^cKit^+^CD34^hi^FcγRII/III^hi^; CMPs, Lin^-^Sca-1^-^cKit^+^CD34^hi^FcγRII/III^low^; MEPs, Lin^-^Sca-1^-^cKit^+^CD34^low^FcγRII/III^low^. For lineage analysis, cells were stained with combinations of Gr1, Mac1, B220, CD4 and CD8 antibodies. For cell cycle analysis, cells were initially stained with antibodies to recognize live hematopoietic cell subpopulations before treatment with Cytofix/Cytoperm buffer kit (BD Biosciences), followed by Ki-67 (1:100, 16A8, 652406, BioLegend) and DAPI staining. Active caspase-3 staining was performed in a similar manner as cell cycle staining, but used anti-active caspase-3 Alexa Fluor 647 (1:50, 560626, BD Biosciences) and fixable viability dye to distinguish active caspase-3 positive and dead cells respectively. An annexin-V apoptosis detection kit (BD Biosciences) was used to evaluate apoptosis according to the manufacturer’s instructions. DAPI staining of ethanol-fixed U937 *CUX1*^*−/−*^ cells, was used to determine cell cycle distribution, whereas BioLegend antibodies to CD11b (ICRF44), CD14 (M5E2), CD34 (581) and CD33 (WM53) were used at 1:100 dilution to characterize the immunophenotype of these cells. Cell sorting was performed on a BD Influx machine (BD Biosciences). Flow cytometric data was acquired using Fortessa or LSR II instruments (BD Biosciences). Data were analyzed using FlowJo software (v10.0.8r1, Tree Star). Gating strategies to identify hematopoietic stem and progenitor cell populations are provided in Supplementary Fig. 11.

### Bone marrow transplantation

For leukemia transplant assays, 2 × 10^5^ red cell-depleted bone marrow cells from *Flt3*^*ITD*^ or *Cux1*^*+/−*^;*Flt3*^*ITD*^ mice were injected into irradiated (5.4 Gy, given as two split doses 4 h apart) CD45.1^+^ B6.SJL-*Ptprc*^*a*^*Pepc*^*b*^/BoyJ mice along with 2 × 10^5^ wild-type CD45.1^+^ bone marrow support cells. Transplanted animals were monitored for disease development by serial blood counts and daily examination. For in vivo *Cflar* knockdown competitive transplant experiments, 10^5^ Tet-pLKO puro shCflar-infected c-Kit^+^ cells from Con, *Cux1*^*+/−*^, *Flt3*^*ITD*^ or *Cux1*^*+/−*^;*Flt3*^*ITD*^ CD45.2^+^ mice were injected with an equal number of CD45.1^+^CD45.2^+^ wild-type bone marrow cells into lethally irradiated of CD45.1^+^CD45.2^+^ recipients. Doxycycline chow containing 625 mg/kg doxycycline (TD.01306, Envigo) was given to the experimental cohort to induce *Cflar* knockdown following establishment of comparable engraftment, whereas the control groups were maintained on normal diet. Bone marrow donor CD45.2 chimerism was distinguished from CD45.1^+^CD45.2^+^ support and recipient cells by flow cytometry two weeks later.

### Immunoblotting

Cells were rinsed in PBS before lysis in RIPA buffer (50 mM Tris [pH 7.4], 150 mM NaCl, 1 mM EDTA, 1% Triton X-100, 0.1% sodium deoxycholate, 0.1% SDS) supplemented with protease/phosphatase inhibitor cocktail (Cell Signaling Technology). Nuclear lysates were prepared according to standard methods. Samples were cleared by centrifugation at 16,000 × *g* at 4 °C for 10 min and protein concentrations were determined using BCA protein assay reagent (Pierce). Equivalent amounts of protein were resolved on 4-12% Bis-Tris or 3-8% Tris-Acetate protein gels (NuPAGE) according to the manufacturer’s instructions and transferred to nitrocellulose membranes (Amersham). Immunoblotting was performed according to standard procedures. The following primary antibodies used at 1:1,000 dilution were purchased from Cell Signaling Technology: anti-HDAC1 (5356); anti-CFLAR (56343); anti-β-Actin (3700); anti-CASP8 (9746); mouse-specific anti-CASP8 (4927); anti-cleaved CASP8 (9496); mouse-specific anti-cleaved CASP8 (8592); anti-PARP (9542); anti-CASP3 (9662); anti-cleaved CASP3 (9661); anti-cleaved PARP (5625); anti-MLKL (37705); anti-p-MLKL^S345^ (37333); anti-XIAP (2042); anti-NFκB p65 (8242); anti- IκBα (4814); anti-NFκB p100/p52 (4882); and anti-vinculin (13901). Other antibodies used at 1:1,000 dilution were: anti-CUX1 (ABE218, Millipore; 11733-1-AP, Proteintech); anti-myc (MA1-980, Invitrogen); anti-cIAP1/2 (MAB3400, Novus); and anti-Vinculin (SAB4200080, Sigma). HRP-linked streptavidin (1:1,000, 3999) and secondary antibodies (1:2,000, anti-rabbit IgG, 7074; 1:2,000, anti-mouse IgG, 7076) were from Cell Signaling Technology. Blots were treated with ECL reagent (Amersham) and signals visualized using an ImageQuant LAS 4000 machine (Amersham) or X-ray film developed with a Compact X4 machine (Xograph). Band quantification was performed using ImageJ (v1.51, NIH).

### Reverse phase protein array (RPPA)

Logarithmically growing cells were collected and washed in PBS before freezing cell pellets at -80 °C. Cell samples and arrays were processed by the RPPA Core at MD Anderson Cancer Center according to published procedures. Post-translationally modified proteins were normalized to total protein levels where possible. Description of sample processing, array methods and data analysis are available on the facility’s website. Three biological replicates per genotype were submitted for analysis.

### Virus production and infection

Retroviral supernatants were produced by transfecting HEK293T cells with transfer and pCL-Ampho or pCL-Eco packaging plasmids. Tet-pLKO-puro, pLKO-GFP, pLKO-mCherry, pLKO.pig, pKLV2-EF1a-Cas9Bsd-W (Addgene, 68343), and pKLV2-based sgRNA lentiviral supernatants were produced by co-transfecting transfer, psPAX2 (Addgene, 12260) and pMD2.G (Addgene, 12259) plasmids into 293FT cells. Lipofectamine 3000 (Invitrogen) was used for transfections according to the manufacturer’s instructions. Viral supernatants were collected 48 h later and frozen at -80 °C. Cell lines were infected with filtered viral supernatants using cell growth media supplemented with 8 µg/ml polybrene (Sigma) for 12-16 h. G418 (1 µg/ml, Gibco) was added 24 h later for MSCV-based infections, whereas puromycin (InvivoGen) was added to 1 µg/ml to select for lentivirus-transduced cells were required.

Mouse c-Kit^+^ cells were isolated from hindlimb bone marrow using CD117 MicroBeads (Miltenyi) and resuspended in StemSpan SFEM media (STEMCELL Technologies) supplemented with glutamine, antibiotics and cytokines (mouse interleukin (IL)-3, 10 ng/ml; mouse IL-6, 10 ng/ml; and mouse stem cell factor (SCF), 25 ng/ml; Peprotech). Cells were transduced by spinoculation at 800 × *g* for 2 h at 32 °C with viral particles previously bound to RetroNectin-coated (Takara) six-well plates. Cells were incubated at 37 °C in 5% CO_2_ for a further 48 h before use in downstream experiments. Puromycin was added to 1 µg/ml to select for transduced cells where required. Doxycycline (Sigma) was added to 1 µg/ml to induce shRNA expression in methylcellulose assays.

Human CD34^+^ bone marrow cells from healthy donors were incubated for two days in StemPro-34 (Gibco) supplemented with StemPro Nutrient Supplement, glutamine, penicillin/streptomycin, human SCF (50 ng/µl), human thrombopoietin (150 ng/µl), human Flt3L (50 ng/µl) and human IL-6 (50 ng/µl) before virus transduction by spinoculation as above. Transduced cells were isolated by sorting for GFP and mCherry expression 48 h later prior to plating into methylcellulose media.

### Methylcellulose colony assays

Murine bone marrow c-Kit^+^ cells were resuspended in IMDM media (Gibco) and added to M3434 methylcellulose media (STEMCELL Technologies) before being dispensed into 35 mm dishes to obtain 500 cells/dish. Cells were incubated at 37 °C in 5% CO_2_ for seven days before scoring colony numbers. Patient-derived hematopoietic and transduced human CD34^+^ cells were mixed with H4435 methylcellulose media (STEMCELL Technologies) and plated into 35 mm dishes with birinapant or drug vehicle as indicated (1-3 × 10^5^ plate for patient-derived cells; 500 cells/plate for human CD34^+^ cells). Plates were incubated for 13 days before staining with 0.1% p-iodonitrotetrazolium violet solution overnight. Plate images were captured and colonies were enumerated using ImageJ software (v1.52b, NIH) or counted manually.

### CRISPR/Cas9 drop-out screen

Wild-type and *CUX1*^*−/−*^ U937 cells were transduced with pKLV2-EF1a-Cas9Bsd-W viral supernatant to generate stable Cas9-expressing cell lines after blasticidin (InvivoGen, 10 µg/ml) selection. Cas9 activity was assessed with GFP reporter assays following transduction of Cas9-expressing cells with pKLV2-U6gRNA5(gGFP)-PGKBFP2AGFP-W or pKLV2-U6gRNA5(Empty)-PGKBFP2AGFP-W lentiviral supernatants. To achieve a 200-fold library representation and a transduction efficiency of ~30% as measured by BFP fluorescence (in order to avoid multiple sgRNAs per cell), 6.1 × 10^7^ *CUX1*-wild-type and *CUX1*^*−/−*^ Cas9-expressing U937 cells were transduced with the Human Improved Genome-wide Knockout CRISPR Library v1 (Addgene, 67989), which contains 90,709 guides targeting 18,010 genes (3-5 sgRNAs per gene). The volume of viral supernatant required for each screen was calculated from preliminary titration experiments. Puromycin (1 µg/ml) was added two days after infection to select for sgRNA-infected cells and cultured for an additional 21 days (around 16 cell doublings in total), ensuring that 45 × 10^6^ cells, representative of ~500 cells per sgRNA, were subcultured to maintain cell densities of 0.25-1.5 × 10^6^ cells/ml. The sgRNAs from transfected *CUX1*^*−/−*^, *CUX1*-wild-type and the sgRNA plasmid library were amplified by PCR from DNA and sequenced as previously described^60^. Adapter-trimmed fastq sequencing reads were directly compared to a list of sgRNA sequences^60^ to quantify the abundance of each sgRNA in each sample. BAGEL v. 0.9^61^ was used to compute the log fold-change of each sgRNA in *CUX1*^*−/−*^ and wild-type replicates compared with the plasmid library at timepoints up to day 21 after drug selection. BAGEL was used to calculate a Bayes Factor (BF) lethality score for each gene at a given timepoint. At the day 21 timepoint we computed a linear model of the gene BF for *CUX1*^*−/−*^ cell lines versus the gene BF for wild-type cell lines. The residuals of the linear model indicate the deviation of the gene-level lethality of the library in the *CUX1*^*−/−*^ compared with the wild-type. Higher residual values suggest that knockout of the target gene in *CUX1*^*−/−*^ cells is significantly more lethal than in wild-type cells. The Z score of each residual was computed as Z_gene = [residual(gene) - mean(all_residuals)] / stddev(all_residuals).

### Competitive sgRNA abundance validation assays

Wild-type or *CUX1*^*−/−*^ U937 or THP-1 cells were infected with lentiviral supernatants to co-express individual sgRNAs targeting *CFLAR* or *AIPL1* (a nonessential gene serving as a control), linked to BFP. The abundance of BFP^+^ cells was determined by flow cytometry over a 20-21-day period in each case. Results were normalized to starting values and BFP^+^ cell abundances in *CUX1*^*−/−*^ cells were calculated relative to wild-type frequencies. Experiments were performed in triplicate.

### Quantitative RT-PCR (qRT-PCR)

Total RNA was extracted using RNeasy Mini or Micro kits (Qiagen) depending on cell input number. Genomic DNA was digested by including an on-column DNase step and RNA integrity was evaluated using a NanoDrop ND-1000 spectrophotometer (Thermo) or an RNA 6,000 Pico kit (Agilent). RNA was reversed transcribed using SuperScript VILO Master Mix (Invitrogen). Quantitative RT-PCR was performed using Taqman Fast Advanced Master Mix (Applied Biosystems) on a QuantStudio 7 machine (Applied Biosystems). Gene expression was normalized to *HPRT1* or *Gapdh* probe expression. Fold expression changes were calculated using the ΔΔCt method. Taqman probes: *HPRT1*, Hs99999909_m1; *CYBB*, Hs00166163_m1; *CFLAR*, Hs01116280_m1; *Cflar*, Mm01255578_m1; *Gapdh*, Mm99999915_g1.

### Chromatin immunoprecipitation (ChIP)

Doxycycline-inducible mCherry, CUX1^p100^ and CUX1^p200^ U937 cells were treated with 1% formaldehyde for 10 min at room temperature, followed by 0.125 M glycine for a further 5 min. Cells were washed in PBS and cell pellets were frozen at -80 °C prior to ChIP experiments. For ChIP, cells were resuspended in lysis buffer (10 mM Tris-HCl [pH 8.0], 10 mM NaCl, 0.2% IGEPAL CA-630, 1 mM PMSF, cOmplete protease inhibitor [Roche]) for 10 min on ice. Nuclei were pelleted by centrifugation and lysed in nuclear buffer (50 mM Tris-HCl [pH 8.0], 10 mM EDTA, 1% SDS, 1 mM PMSF, cOmplete protease inhibitor [Roche]). DNA was sheared with seven cycles 30 s on/off using a Bioruptor Pico device (diagenode). The soluble chromatin fraction was recovered and diluted with dilution buffer (20 mM Tris-HCl [pH 8.0], 150 mM NaCl, 2 mM EDTA, 1% Triton X-100, 0.01% SDS, 1 mM PMSF, cOmplete protease inhibitor [Roche]). Samples were incubated with Dynabeads MyOne Streptavidin T1 (Invitrogen), previously blocked with 1% fish gelatin (Sigma), overnight at 4 °C. The following morning, beads were washed with 2% SDS, followed by wash 1 (50 mM HEPES, pH 7.5, 500 mM NaCl, 1 mM EDTA, 1% Triton X-100, 0.1% deoxycholic acid), wash 2 (10 mM Tris-HCl, pH 8.0, 250 mM LiCl, 1 mM EDTA, 0.5% IGEPAL CA-630, 0.5% deoxycholic acid) and TE buffer (10 mM Tris-HCl, pH 7.5, 1 mM EDTA). DNA was eluted from beads using elution buffer (50 mM Tris-HCl, pH 8.0, 1% SDS, 10 mM EDTA) at 65 °C overnight with agitation using a ThermoMixer (Eppendorf). Samples were treated with RNase A (Invitrogen) and proteinase K (Ambion) before recovery of genomic DNA using phenol chloroform. ChIP qRT-PCR was performed using PowerUp SYBR Green Master Mix (Applied Biosystems) on a QuantStudio 7 machine (Applied Biosystems). The percentage input method was used to calculate DNA recovery of specific genomic loci. Primers corresponding to the CFLAR promoter were CFLARChF1 and CFLARChR1. Primers annealing to a distal control genomic region were CFLAR_CHIPconF and CFLAR_CHIPconR.

### Proliferation and drug treatment assays

1 × 10^4^ U937 *CUX1*^*−/−*^ or wild-type cells were dispensed into 96 well plates in triplicate. Cytarabine (Apexbio) was dissolved in water. Drug or vehicle were added to cells to achieve the desired concentrations as indicated. Cells were incubated for 48 h before cell viability was determined using CellTiter 96 AQ_ueous_ One reagent (Promega). For birinapant treatment of murine cKit^+^ cells, 2 × 10^4^ cells were plated in 96 well plates containing RPMI supplemented with 10% FBS, glutamine, antibiotics and cytokines (mouse IL-3, 10 ng/ml; mouse IL-6, 10 ng/ml; and mouse SCF, 25 ng/ml; Peprotech) in triplicate from three mice per genotype. Birinapant (Selleckchem) or DMSO vehicle was added to achieve desired concentrations and a final well volume of 200 µl before incubating for a further 48 h. Proliferation assays using murine cKit^+^ cells infected with *Cflar* shRNA vectors were performed similarly using 2 × 10^4^ cells plated in 96 well plates in triplicate from two mice per genotype with doxycycline (Sigma) or drug vehicle added to 0.1 µg/ml. Apoptosis was determined by Annexin-V and DAPI staining. Necrostatin-1 (20 µM, Sigma) or zVAD (10 µM, Selleckchem) was added at the same time as birinapant or doxycycline where indicated in the text.

### Plasmids, shRNAs and siRNA

Lentiviral transfer plasmids expressing sgRNAs targeting *CFLAR*, and *AIPL1* genes were generated by cloning annealed sgRNA oligonucleotides (CFLAR1_For, CFLAR1_Rev; and AIPL1_For, AIPL1_Rev), into the BbsI site of pKLV-U6gRNA(BbsI)-PGKpuro2ABFP (Addgene, 50946), leading to pKLV-U6gRNA(CFLAR1)-PGKpuro2ABFP and pKLV-U6gRNA(AIPL1)-PGKpuro2ABFP plasmids respectively. For CRISPR/Cas9-resistant *CFLAR* complementation experiments, CFLAR2_For and CFLAR2_Rev oligonucleotides were annealed and cloned into of pKLV-U6gRNA(BbsI)-PGKpuro2ABFP, leading to pKLV-U6gRNA(CFLAR2)-PGKpuro2ABFP. Lentiviral sgRNA transfer plasmids targeting *CUX1* and *ACCSL* genes were generated by cloning annealed sgRNA oligonucleotides (CUX1crsp9_For, CUX1crsp9_Rev; and ACCSL_For, ACCSL_Rev), into the BbsI site of pKLV2-U6gRNA5(BbsI)-PGKpuro2AmCherry-W (Addgene, 67977), leading to pKLV2-U6gRNA5(CUX1)-PGKpuro2AmCherry-W and pKLV2-U6gRNA5(ACCSL)-PGKpuro2AmCherry-W plasmids respectively. The non-essential *AIPL1* and *ACCSL* genes were used as control sgRNA targets for CRISPR/Cas9 experiments. For *CUX1* ablation in U937 cells, two sgRNA sequences, annealed CUX1crsp8 (CUX1crsp8_For and CUX1crsp8_Rev) and CUX1crsp9 (CUX1crsp9_For and CUX1crsp9_Rev) oligonucleotides were cloned into pX458 (Addgene, 48138) which target exon 18 of *CUX1*, to generate sgRNA-expressing vectors pX458 CUX1crsp8 and pX458 CUX1crsp9 respectively. Relevant portions of all plasmids were verified by DNA sequencing or restriction digests. Enzymes were purchased from New England Biolabs.

To create doxycycline-regulatable vectors expressing epitope-tagged CUX1^p110^ or CUX1^p200^ that are substrates for biotin holoenzyme synthetase (BirA), CUX1^p110^ or CUX1^p200^ coding sequences were subcloned from pXJ myc-CUX1^p110^-HA and pXJ myc-CUX1^p200^-HA into pKS Bio-FLAG so that CUX1 isoforms were N- and C-terminally tagged with biotinylatable peptides and HA tags respectively. Epitope-tagged CUX1 proteins or mCherry were subsequently subcloned downstream of a tetracycline response element (TRE) in PB BirA-P2A-rtTA-EGFP-TRE which also harbors: BirA, rtTA and EGFP coding sequences separated by P2A motifs under the control of a CAGGS promoter; and flanking Piggybac inverted terminal repeats. PB BirA-P2A-rtTA-EGFP-TRE mCherry, PB BirA-P2A-rtTA-EGFP-TRE CUX1^p110^ and PB BirA-P2A-rtTA-EGFP-TRE CUX1^p200^ plasmids were verified by sequencing.

For human CFLAR expression, an IRES-EGFP cassette from pIRES2-EGFP (Clontech) was firstly cloned downstream of the neomycin-resistance gene in MSCVneo (Clontech) to create MSCVneo IRES-EGFP. The *CFLAR* coding sequence with a C-terminal V5 tag was cloned into MSCVneo IRES-EGFP, leading to MSCVneo IRES-EGFP CFLAR-V5. For mouse *Cflar* expression, the mouse *Cflar* ORF was amplified from IMAGE clone 4460554 using mCflarHpaF and mCflarSalR by PCR. The PCR product was cut with HpaI and SalI and cloned into MSCV IRES GFP which had been cut with the same enzymes, creating MSCV mCflar IRES EGFP.

To construct *CFLAR* promoter-luciferase vectors, the genomic region upstream of the *CFLAR* transcriptional start site was amplified by PCR using CFLARpr_KpnRVFor and CFLARpr_XhNhRev. The PCR product was digested with NheI, blunted with Klenow and cut subsequently with KpnI prior to cloning in pGL4.10[luc2] (Promega), which had been digested with HindII, treated with Klenow and cut subsequently with KpnI, thereby creating pGL4.10-CFLARprom^WT^. *CFLAR* promoter-luciferase deletion constructs were made as follows from pGL4.10-CFLARprom^WT^: pGL4.10-CFLARprom^∆1^, EcoRV and BglII digest; pGL4.10-CFLARprom^∆2^, HpaI-XhoI fragment was cloned into EcoRV and XhoI-digested pGL4.10-CFLARprom^WT^; pGL4.10-CFLARprom^∆3^, EcoRV and HindIII digest; pGL4.10-CFLARprom^∆4^, HindIII and XhoI digest. DNA ends were blunted with Klenow before religating vectors. pGL4.10-CFLARprom^∆5^ was created from pGL4.10-CFLARprom^∆1^ by KpnI and EcoRI digestion before Klenow treatment and religation. The putative CUX1-binding sites in pGL4.10-CFLARprom^∆5^ was mutated by site-directed mutagenesis with Phusion DNA polymerase using CFLAR_mutF and CFLAR_mutR, leading to pGL4.10-CFLARprom^∆5MUT^. To create doxycycline-regulatable *Cflar* shRNA vectors, two pairs of *Cflar*-targeting oligonucleotides (Cflar_sh1For, Cflar_sh1Rev; and Cflar_sh2For, Cflar_sh2Rev) were annealed and cloned into Tet-pLKO-puro (Addgene, 21915). For *CUX1* shRNA knockdown in U937 and THP-1 cells, pLKO.pig shCon and pLKO.pig shCUX1 were used.^18^ To create *CUX1* and *CFLAR* shRNA vectors for human CD34^+^ experiments, which co-express GFP or mCherry, two pairs of *CUX1*-, *CFLAR*- or control-targeting oligonucleotides (CUX1.1: CUXsh1F/CUXsh1R, CUX1.2: CUX1sh2F/CUXsh2R; CFLAR1.2: CFLARshf1/CFLARshf2, CFLAR1.2: CFLARshR1/CFLARshR2; Control: ConF/R) were annealed and cloned into pLKO-GFP or pLKO-mCherry in which the puromycin resistance gene was replaced by the relevant fluorescent protein-coding sequence.

### *CFLAR* promoter-luciferase assay

Promoter-luciferase assays were conducted using HEK293T cells as previously described^18^. HEK293T cells were transfected with Lipofectamine 2000 (Invitrogen) and luciferase activity was determined 24 later. Triplicate transfections were performed in each of three experiments. Data for each promoter construct were normalized to signals derived from pXJ42 empty vector controls.

### Electrophoretic mobility shift assay

Nuclear lysates were prepared from HEK293T cells transfected with pXJ42 or pXJ myc-CUX1^p200^-HA vectors using lipofectamine 2000 (Invitrogen) according the manufacturer’s instructions. Complementary oligonucleotides CFLAR_emsaF1 and CFLAR_emsaR1 representing putative -132 to -122 CUX1-binding sites were annealed and end-labeled using [γ-^32^P] ATP (Perkin Elmer) with T4 polynucleotide kinase (New England Biolabs). Labeled double-stranded DNA probes were prepared in the same way using CFLAR_emsaF2 and CFLAR_emsaR2 oligonucleotides corresponding to potential -156 to -146 CUX1-binding sites. Unincorporated nucleotides were removed using illustra MicroSpin G-25 columns (GE Healthcare) according to the manufacturer’s instructions. Binding reactions were performed by mixing 2 µg nuclear lysate in 20 µl final volume containing binding buffer [10 mM Tris (pH 7.5), 25 mM NaCl, 1 mM MgCl_2_, 5 mM EDTA (pH 8.0), 1 mM DTT and 5% glycerol], 100 ng poly (dI-dC) (Thermo), 3 µg bovine serum albumin (Sigma) and 0.2 pmol radiolabeled probe for 15 min at room temperature. Cold probe competition assays were performed by adding 10-fold molar excess of unlabeled DNA probe 10 min prior to adding the radiolabeled probe. Antibody inhibition assays were performed by preincubating nuclear lysates with normal mouse IgG (12-371, Upstate) or anti-myc antibody (05-724, Upstate). Reactions were separated on 6% DNA retardation gels (Novex) in 0.5 × TBE buffer at 80 V. Gels were dried and exposed to Hyperfilm MP films (Amersham), which were developed using a Compact X4 machine (Xograph).

### Cell culture

THP-1 cells were sourced from the Sanger Cell Lines Project (https://cancer.sanger.ac.uk/cell_lines), whereas U937 cells originated from the European Collection of Authenticated Cell Cultures. Cells were authenticated by short tandem repeat genotyping and verified to be free from mycoplasma contamination. Cells were grown at 37 °C in 5% CO_2_ using RPMI media supplemented with 10% fetal bovine serum (FBS, Gibco), glutamine, penicillin and streptomycin. HEK293T and 293FT (Invitrogen) cells were grown in DMEM media with 10% FBS, glutamine, penicillin and streptomycin.

### *CUX1* knockout and CUX1-tagged cell line generation

U937 *CUX1*^*−/−*^ cells were generated by electroporating 2.5 µg of pX458 CUX1crsp8 and pX458 CUX1crsp9 plasmids (which express Cas9, an sgRNA targeting *CUX1* exon 18 and EGFP) into 5 × 10^5^ cells in a 100 µl Neon tip using a Neon transfection system (pulse voltage 1400 V, pulse width 10 ms, pulse number 3) according to the manufacturer’s instructions (Thermo). Forty-eight hours later, single EGFP^+^ cells were isolated by flow cytometric sorting into 96-well plates and cultured. *CUX1*^*−/−*^ clones were identified by PCR genotyping and DNA sequencing of *CUX1* exon 18 using primers hCUXsurvF18 and hCUXsurvR18, followed by immunoblotting for CUX1 protein.

Doxycycline-regulatable mCherry, CUX1^p110^ and CUX1^p200^ expression in U937 *CUX1*^*−/−*^ cells was achieved by electroporating 2.5 µg pCMV-hyPBase along with 2.5 µg PB BirA-P2A-rtTA-EGFP-TRE mCherry, PB BirA-P2A-rtTA-EGFP-TRE CUX1^p110^ or PB BirA-P2A-rtTA-EGFP-TRE CUX1^p200^ into U937 *CUX1*^*−/−*^ cells using a Neon transfection system. EGFP^+^ cells were isolated by flow cytometric sorting and expanded. Protein expression was induced by adding doxycycline (0.1 µg/ml) to the growth media 24-36 h before cell harvest.

To generate THP-1 *CUX1*^*−/−*^ and control sgRNA-expressing cell lines, THP-1 cells were first infected with Cas9-expressing pKLV2-EF1a-Cas9Bsd-W lentivirus and selected with blasticidin. THP-1:Cas9 cells were subsequently infected with lentiviral supernatants produced using pKLV2-U6gRNA5(CUX1)-PGKpuro2AmCherry-W or pKLV2-U6gRNA5(ACCSL)-PGKpuro2AmCherry-W transfer plasmids and selected with puromycin.

### RNA sequencing

Total RNA was extracted from sorted LSK mouse cells using RNeasy Micro kits with on column DNA digestion (Qiagen). A SMART-Seq v4 Ultra Low Input RNA kit (Clontech) was used to synthesize cDNA libraries from LSK cells. Total RNA samples were evaluated using an RNA 6,000 Pico kit (Agilent). Indexed sequencing libraries from LSK cDNA samples were prepared using a Nextera XT DNA library preparation kit (Illumina). Libraries were pooled and sequenced using an Illumina HiSeq 2000 system to produce 75 bp paired-end reads for LSK cells. For mouse transcriptome data analysis, adapter-trimmed sequences were mapped to the GRCm38 assembly with STAR (v2.5.0)^62^. Gene annotations were from Ensembl release 84. Gene-level quantification was performed using HTSeq (v.0.7.2) (ref. ^63^). Differential gene expression between control and experimental samples was determined using DESeq2 package^64^ (v.1.24.0). GSEA (v4.0.3) was performed using curated gene sets from the Molecular Signatures Database and studies relevant to hematopoietic self-renewal and differentiation signatures^65^. DAVID (v6.8) was used for functional gene annotation^66^.

### Statistical analyses

Two-tailed, unpaired *t*-tests or Mann-Whitney tests were performed to assess statistical significance in pairwise comparisons. Log-rank (Mantel-Cox) tests were performed on survival curves to determine significant differences. One-way or two-way ANOVA with Tukey’s or Dunnett’s tests were used for multiple group comparisons.

### Reporting summary

Further information on research design is available in the Nature Research Reporting Summary linked to this article.

## Supplementary information

Supplementary Information

Description of Additional Supplementary Files

Supplementary Data 1-6

Reporting Summary

## Data Availability

The mouse LSK RNA-sequencing data for this study have been deposited in the European Nucleotide Archive (ENA) at EMBL-EBI under accession number P1. Molecular Signatures Database v7.2 is available through GSEA website (https://www.gsea-msigdb.org/gsea/msigdb/index.jsp). The source data underlying Figs. 1–8 and Supplementary Figures are provided as a Source Data file. All the other data supporting the findings of this study are available within the article and its supplementary information files and from the corresponding author upon reasonable request. Source data are provided with this paper.

## Source data

Source Data
