# Supplementary material for: Cut-like homeobox 1 (CUX1) tumor suppressor gene haploinsufficiency induces apoptosis evasion to sustain myeloid leukemia
Source: Nat Commun. 2021 Apr 30;12:2482. doi: 10.1038/s41467-021-22750-8 (PMC8087769; doi:10.1038/s41467-021-22750-8)
Supplement: Supplementary file 1 — Supplementary Information [file 41467_2021_22750_MOESM1_ESM.pdf]

Supplementary Information

Cut-like homeobox (*CUX1*) tumor suppressor gene haploinsufficiency induces apoptosis evasion to sustain myeloid leukemia

Supper *et al*.

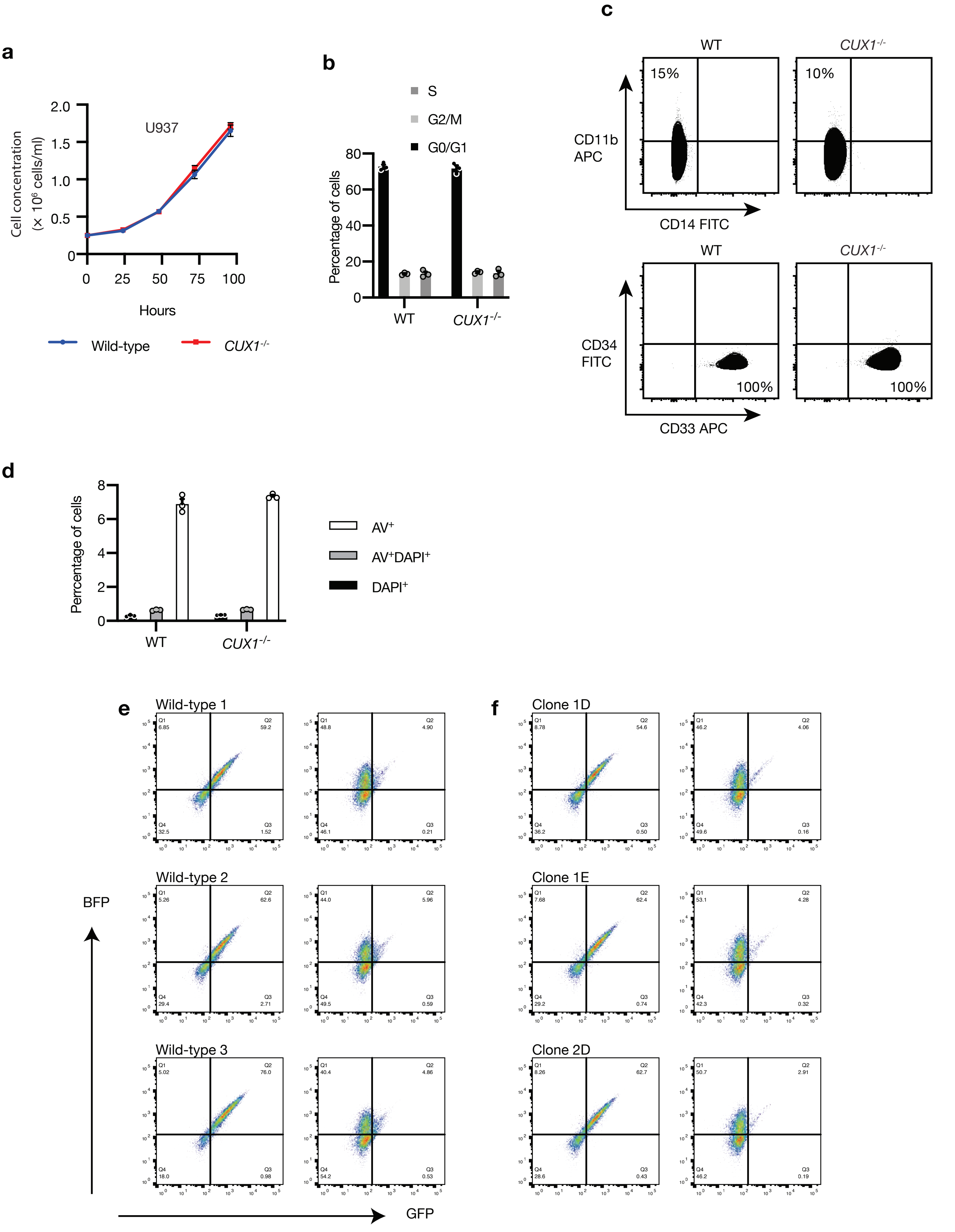

Supplementary Figure 1

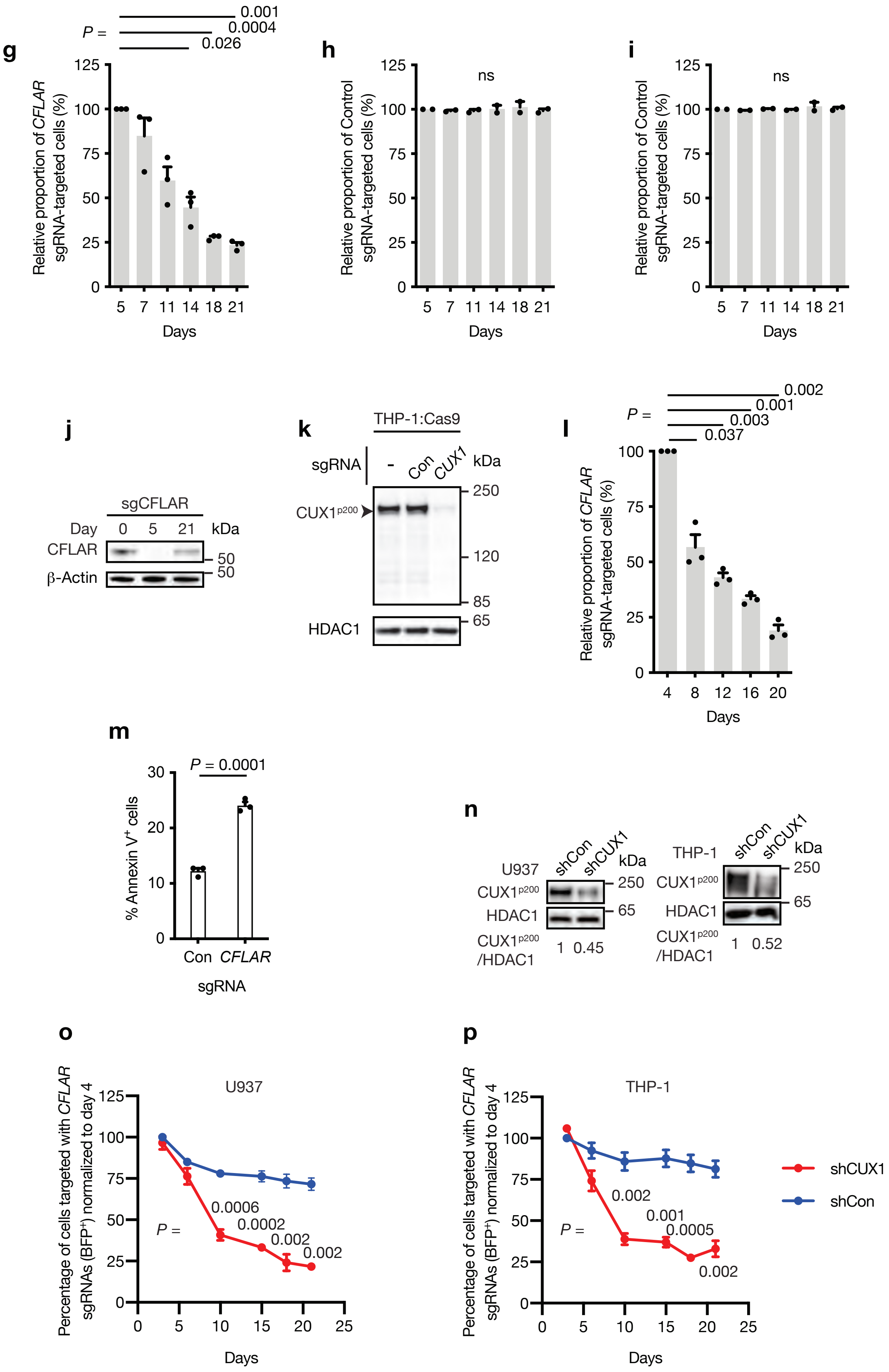
g
P =
0.001
0.0004
0.026
Relative proportion of CFLAR sgRNA-targeted cells (%)
125
100
75
50
25
0
5 7 11 14 18 21
Days
h
ns
Relative proportion of Control sgRNA-targeted cells (%)
125
100
75
50
25
0
5 7 11 14 18 21
Days
i
ns
Relative proportion of Control sgRNA-targeted cells (%)
125
100
75
50
25
0
5 7 11 14 18 21
Days
j
sgCFLAR
Day 0 5 21 kDa
CFLAR
β-Actin
50
50
k
THP-1:Cas9
sgRNA - Con CUX1 kDa
CUX1p200
250
120
85
HDAC1
65
l
P =
0.002
0.001
0.003
0.037
Relative proportion of CFLAR sgRNA-targeted cells (%)
100
75
50
25
0
4 8 12 16 20
Days
m
P = 0.0001
% Annexin V+ cells
30
20
10
0
Con CFLAR
sgRNA
n
U937
shCon shCUX1 kDa
CUX1p200
250
HDAC1
65
CUX1p200/HDAC1
1 0.45
THP-1
shCon shCUX1 kDa
CUX1p200
250
HDAC1
65
CUX1p200/HDAC1
1 0.52
o
U937
Percentage of cells targeted with CFLAR sgRNAs (BFP+) normalized to day 4
125
100
75
50
25
0
P =
0.0006
0.0002
0.002
0.002
0 5 10 15 20 25
Days
p
THP-1
Percentage of cells targeted with CFLAR sgRNAs (BFP+) normalized to day 4
125
100
75
50
25
0
P =
0.002
0.001
0.0005
0.002
0 5 10 15 20 25
Days
shCUX1
shCon

Supplementary Figure 1. Characterization of *CUX1*$^{-/-}$ U937 cells and validation of *CFLAR* as a genetic vulnerability in *CUX1*$^{-/-}$ cell lines

(**a**) Plot showing growth of wild-type and *CUX1*$^{-/-}$ U937 cells. Cells were seeded in triplicate at 2.5×10^6 cells/ml and the growth of each sample was measured daily for four days. Doubling times were comparable: wild-type, 32.7 h; *CUX1*$^{-/-}$; 32.4 h.

(**b**) Comparable cell cycle distribution of wild-type (WT) and *CUX1*$^{-/-}$ U937 cells. The percentage of cells in each cell cycle phase is shown. Measurements from three independent samples were taken.

(**c**) Flow cytometry plots showing comparable immunophenotype of wild-type (WT) and *CUX1*$^{-/-}$ U937 cells. Cells were stained with stem cell (CD34) and myelomonocytic markers (CD33, CD11b, CD14).

(**d**) Bar plot showing the percentage of apoptotic cells by Annexin-V/DAPI staining in wild-type and *CUX1*$^{-/-}$ U937 cells. Measurements from three independent samples were taken. *CUX1*$^{-/-}$ U937 clone 1D was used in assays (a)-(d).

(**e,f**) Flow cytometric plots of three Cas9-expressing wild-type (e) and three Cas9-expressing *CUX1*$^{-/-}$ (f) U937 cell lines showing expression of BFP and GFP. Left panels show fluorescence patterns following cell infection with GFP- and BFP-expressing lentivirus. Right panels show comparable loss of GFP fluorescence between cell lines following cell infection with a viral vector expressing a GFP-targeting sgRNA along with GFP and BFP.

(**g**) Cas9-expressing *CUX1*$^{-/-}$ (clone 2D) and wild-type cells were infected with viral supernatant to co-express *CFLAR*-targeting sgRNA and BFP. The proportion of *CFLAR* sgRNA-targeted cells in *CUX1*$^{-/-}$ cells was monitored over time. Results were normalized to day 5, which was designated 100%, and to wild-type cells at each time point. Points represent measures from biological replicates.

(**h,i**) Competitive sgRNA depletion assays using BFP-linked control sgRNAs in two *CUX1*$^{-/-}$ cell clones, 1D (h) and 2D (i), reveal no fall in the proportions of cells infected with Control-targeting sgRNA over time. Results were normalized to day 5, which was designated 100%, and to wild-type cells at each time point. Points represent measures from biological replicates.

(**j**) Immunoblot showing changes in levels of CFLAR protein in U937 *CUX1*$^{-/-}$ cells after infection (Day 0) with *CFLAR*-targeting sgRNA. β-Actin was used as loading control. CFLAR is not detectable five days post infection, but is visible again at day 21.

(**k**) Representative immunoblot (n = 2) showing loss of CUX1 expression in THP-1 cells expressing Cas9 and *CUX1*-targeting sgRNA. Cells which neither express any sgRNA (-) or an sgRNA targeting a control gene were used as controls. HDAC1 was used as loading control.

(**l**) Competitive sgRNA assay showing preferential loss of *CFLAR* sgRNA-targeted cells in *CUX1*$^{-/-}$ THP-1 cells compared with wild-type over 20 days. Points represent measures from biological replicates.

(**m**) Increase in Annexin-V^{+} apoptotic cells following *CFLAR*-sgRNA infection compared with control sgRNA (Con) in *CUX1*$^{-/-}$ THP-1 cells. Points represent measures from biological replicates.

(**n**) Representative immunoblot (n = 2) showing levels of CUX1 in U937 (left) and THP-1 (right) cells expressing non-targeting (shCon) or *CUX1*-targeting (shCUX1) shRNAs. HDAC1 was used as loading control. The ratio of CUX1:HDAC1 levels normalized to shCon cells is shown below the blot.

(**o,p**) Competitive sgRNA depletion assays using CFLAR-targeting sgRNAs. U937 (o) and THP-1 (p) cells expressing shCon (blue) or shCUX1 (red) shRNAs were transduced with Cas9-expressing lentivirus. Stable, selected cell lines were subsequently infected with *CFLAR*-targeting sgRNA vectors which co-express BFP. The proportion of *CFLAR* sgRNA-targeted (BFP^{+}) cells was measured over time in three independent experiments and normalized to the start of the assay. Plots show mean + s.e.m.; one-way ANOVA with Dunnett's test for multiple comparisons and repeated measures (g-i, l), two-tailed, unpaired *t*-test (m,o,p).

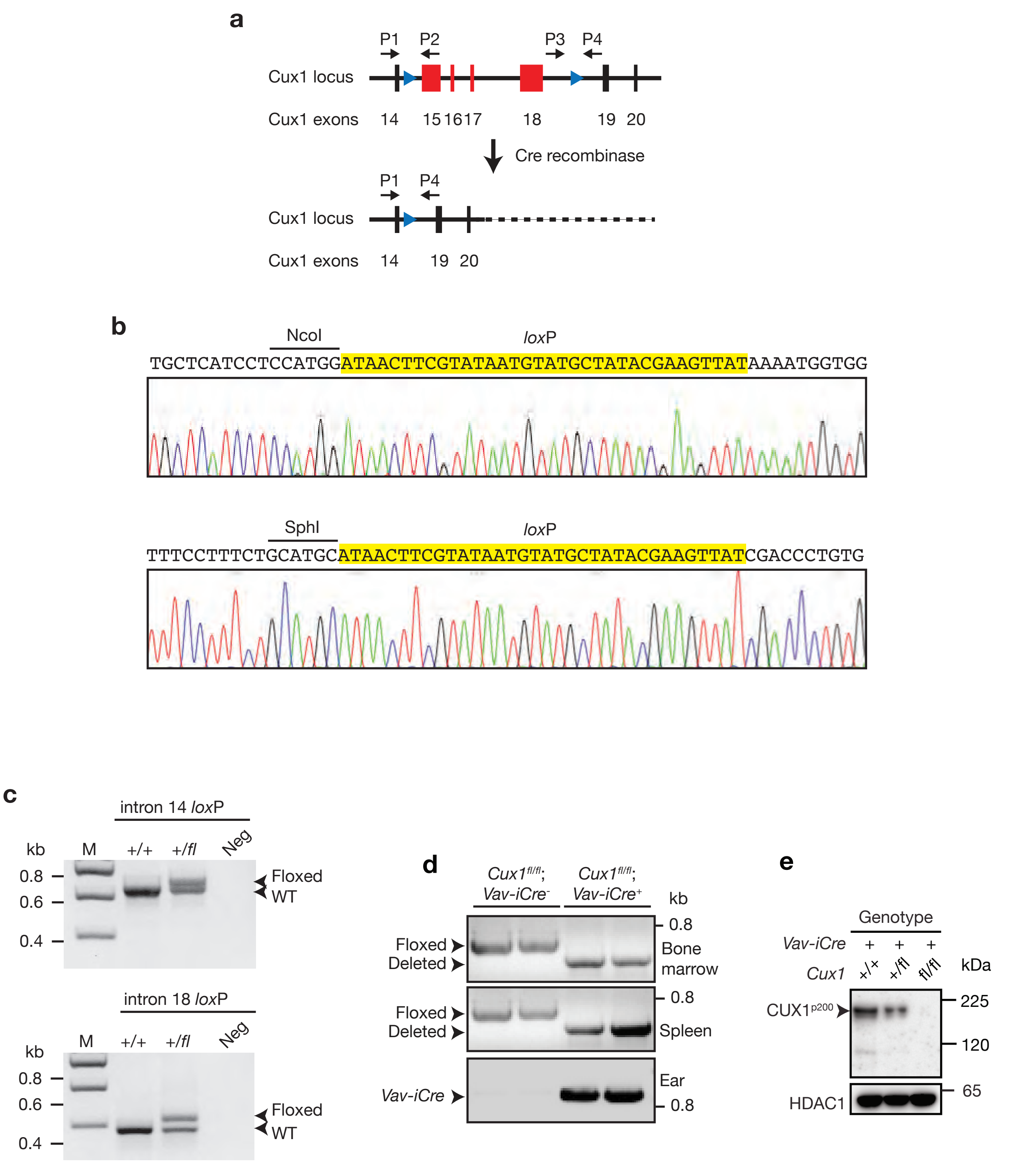

Supplementary Figure 2. Generation and characterization of conditional *Cux1* knockout mice.
(**a**) Schematic of targeted *Cux1* locus (top) showing exons (numbered boxes; red, floxed exons), *lox*P sites (blue triangles) and location of genotyping primers (P1-P4). Anticipated deleted *Cux1* allele following Cre recombinase expression (bottom).
(**b**) DNA chromatograms showing correct *lox*P incorporation (yellow) into Cux1 introns 14 (top) and 18 (bottom) in *Cux1*$^{+/fl}$ mice.
(**c**) Agarose gel image showing PCR genotyping for the *Cux1*^{fl} allele in the founder mouse. M, DNA marker; Neg, no DNA template. Genotyping has been performed several dozen times with results as anticipated.
(**d**) PCR genotyping using tissue DNA showing *Vav-iCre*-associated *Cux1* deletion in bone marrow and spleen from four mice of the indicated genotypes. Genotyping has been performed several dozen times with results as anticipated.
(**e**) Verification of *Vav-iCre*-dependent ablation of CUX1 protein expression by immunoblotting of splenocyte lysates. HDAC1 was used as a loading control. The experiment was performed twice.

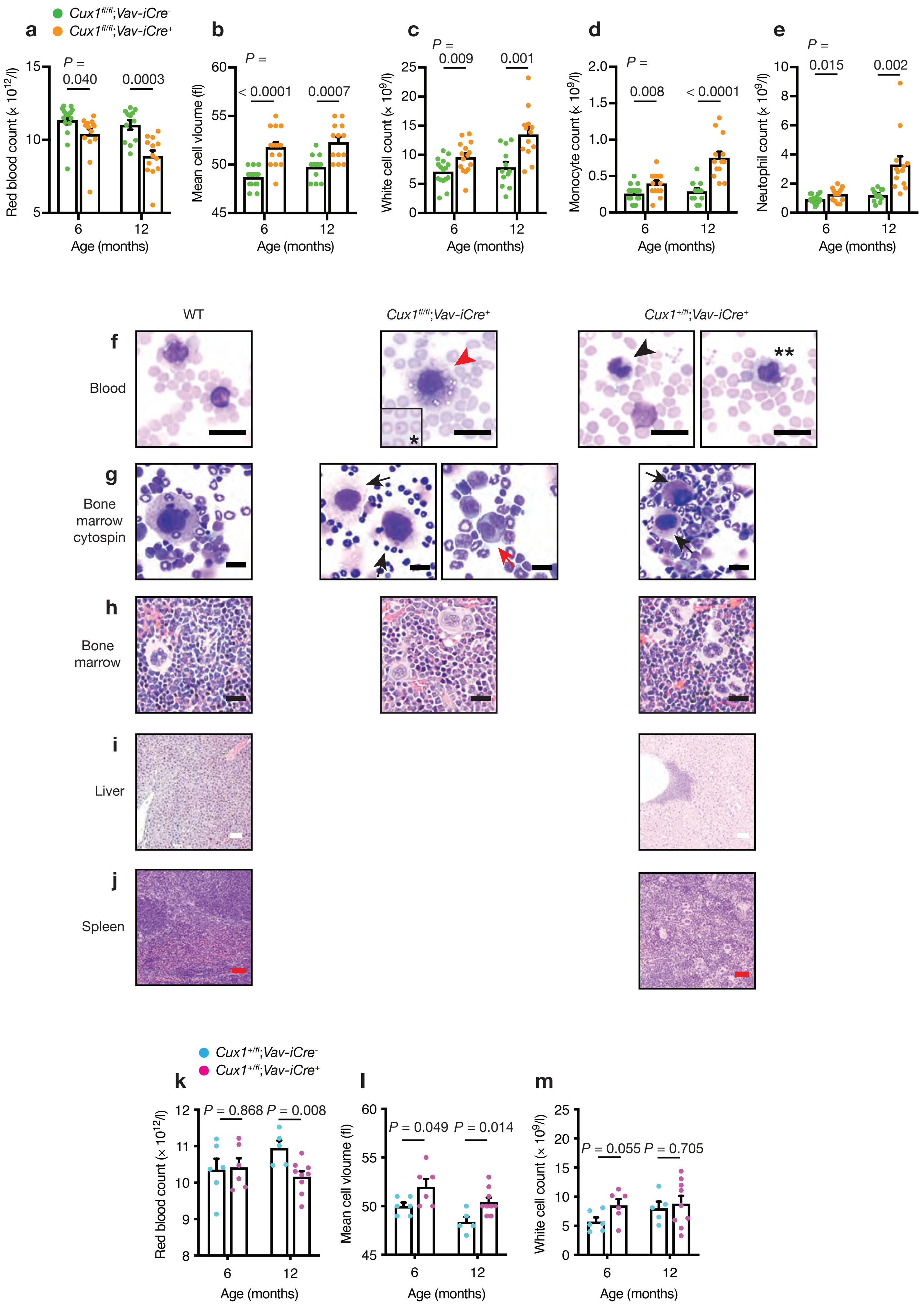

Supplementary Figure 3

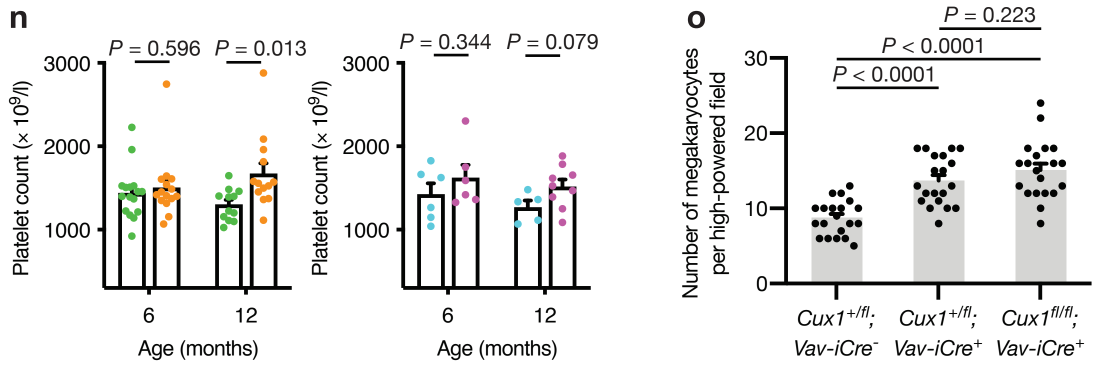

Supplementary Figure 3. Hematologic abnormalities in *Cux1*-deficient mice.
(**a-e**) Red blood count (a) showing anemia, red blood cell mean cell volume (b) showing macrocytosis, white blood count (c) showing leukocytosis, monocyte count (d) showing monocytosis and neutrophil count (e) showing neutrophilia in *Cux1*$^{fl/fl}$;*Vav-iCre*$^{+}$ mice (orange circles) compared with *Cre*-negative littermates (green circles). More severe changes are observed in older mice. *Cux1*$^{fl/fl}$;*Vav-iCre*$^{-}$, n = 15-16; *Cux1*$^{fl/fl}$;*Vav-iCre*$^{+}$ n = 12-13.
(**f,g**) Representative May-Grünwald-Giemsa-stained blood (f) and bone marrow (g) smears from mice of the indicated genotypes. Black arrowhead, bilobed neutrophil; red arrowhead, circulating hypolobated megakaryocyte; asterisk, Howell Jolly body; double asterisks, immature monocyte; black arrows, hypolobated megakaryocytes; red arrows, bilobed erythroid cells. Five mice per genotype were assessed.
(**h-j**) Representative hematoxylin and eosin-stained bone marrow (h), liver (i) and spleen (j) sections from mice of the indicated genotypes. Note frequent dysplastic megakaryocytes in bone marrow of *Cux1*-deficient mice. The livers are infiltrated with myelomonocytic cells and the splenic architecture is effaced with red pulp expansion containing numerous megakaryocytes and myelomonocytic cells. Scale bars; black = 20 μm, white = 100 μm, red = 50 μm. WT, wild-type. Five mice per genotype were assessed.
(**k-m**) Red blood count (k) showing anemia, red blood cell mean cell volume (l) showing macrocytosis in *Cux1*$^{+/}$*fl*;*Vav-iCre*$^{+}$ mice (magenta circles) compared with *Cre*-negative littermates (cyan circles). Significant anemia is detected at 12 months of age, whereas macrocytosis is observed earlier on. There was no difference in white cell counts (m). *Cux1*$^{+/fl}$;*Vav-iCre*$^{-}$, n = 6; *Cux1*$^{+/fl}$;*Vav-iCre*$^{+}$, n = 9.
(**n**) Platelet counts in *Cux1*$^{fl/fl}$;*Vav-iCre*$^{+}$ (left) and *Cux1*$^{+/fl}$;*Vav-iCre*$^{+}$ mice (right) compared with corresponding controls at six and 12 months of age. Platelet counts are higher at 12 months of age in *Cux1*$^{fl/fl}$;*Vav-iCre*$^{+}$ mice, but less so in *Cux1*$^{+/fl}$;*Vav-iCre*$^{+}$ mice.
(**o**) Plot showing number of megakaryocytes per high-powered field in mice of indicated genotypes. The number of megakaryocytes was determined by examining five fields (magnification × 400) in four mice per genotype. Each circle represents one mouse or field. All plots show mean + s.e.m; two-tailed, unpaired *t*-test (a-e, k-o).

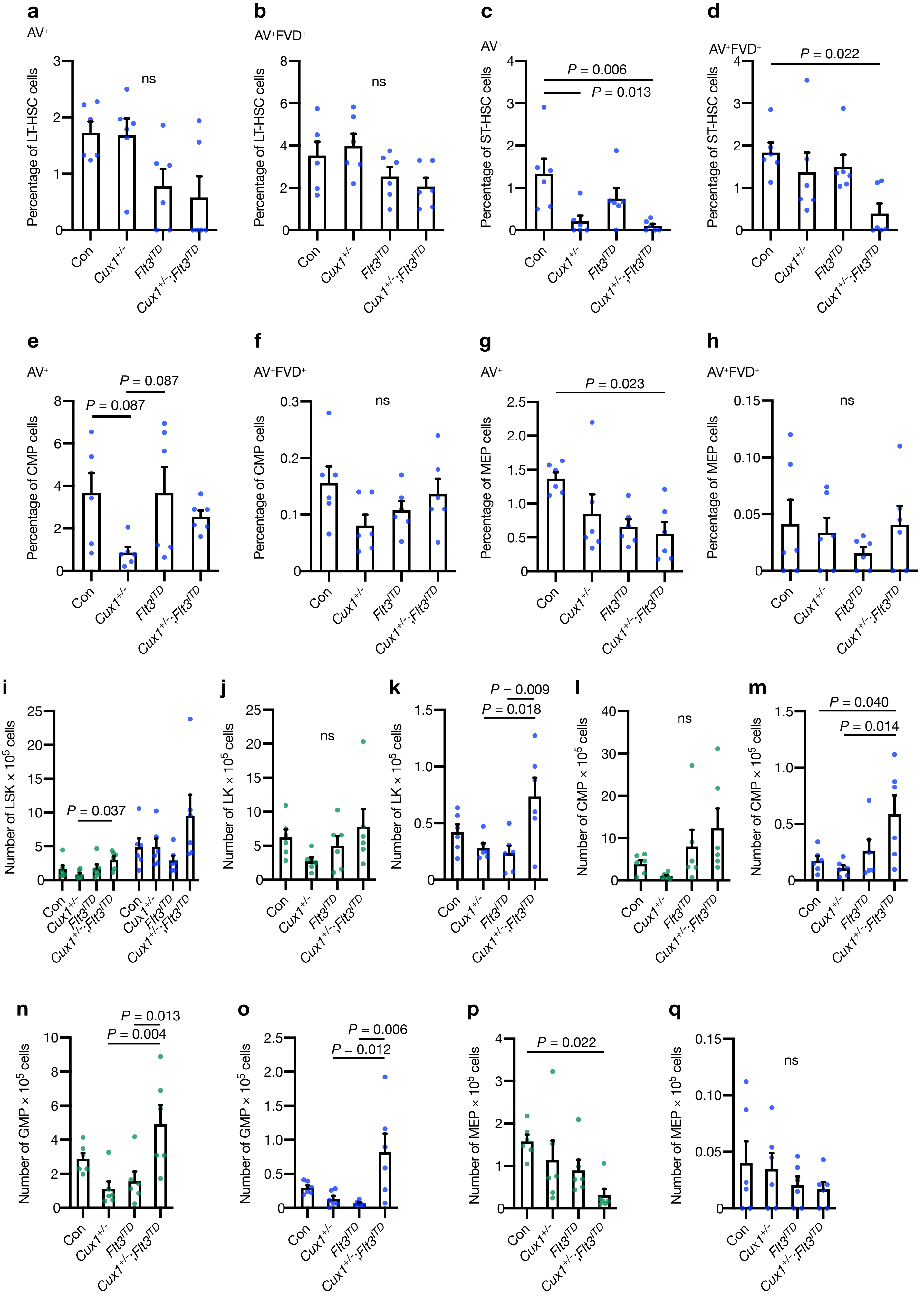

Supplementary Figure 4

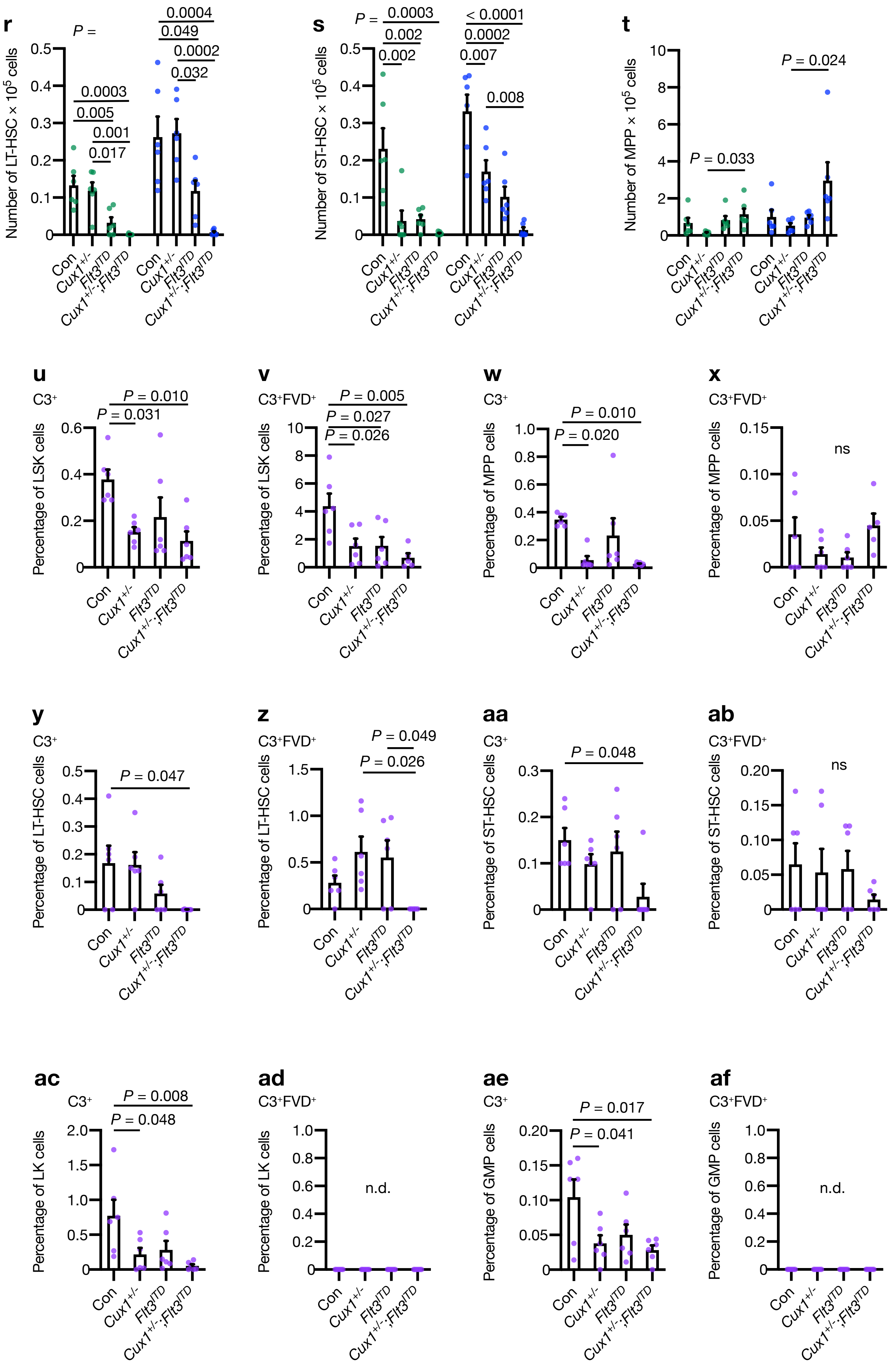

r
Number of LT-HSC × 10^5 cells
P =
0.0004
0.049
0.0002
0.032
0.0003
0.005
0.001
0.017
Con
Cux1+/-
Flt3ITD
Cux1+/-;Flt3ITD
s
Number of ST-HSC × 10^5 cells
P = 0.0003
0.002
0.002
< 0.0001
0.0002
0.007
0.008
t
Number of MPP × 10^5 cells
P = 0.033
P = 0.024
u
C3+
Percentage of LSK cells
P = 0.010
P = 0.031
v
C3+FVD+
Percentage of LSK cells
P = 0.005
P = 0.027
P = 0.026
w
C3+
Percentage of MPP cells
P = 0.010
P = 0.020
x
C3+FVD+
Percentage of MPP cells
ns
y
C3+
Percentage of LT-HSC cells
P = 0.047
z
C3+FVD+
Percentage of LT-HSC cells
P = 0.049
P = 0.026
aa
C3+
Percentage of ST-HSC cells
P = 0.048
ab
C3+FVD+
Percentage of ST-HSC cells
ns
ac
C3+
Percentage of LK cells
P = 0.008
P = 0.048
ad
C3+FVD+
Percentage of LK cells
n.d.
ae
C3+
Percentage of GMP cells
P = 0.017
P = 0.041
af
C3+FVD+
Percentage of GMP cells
n.d.

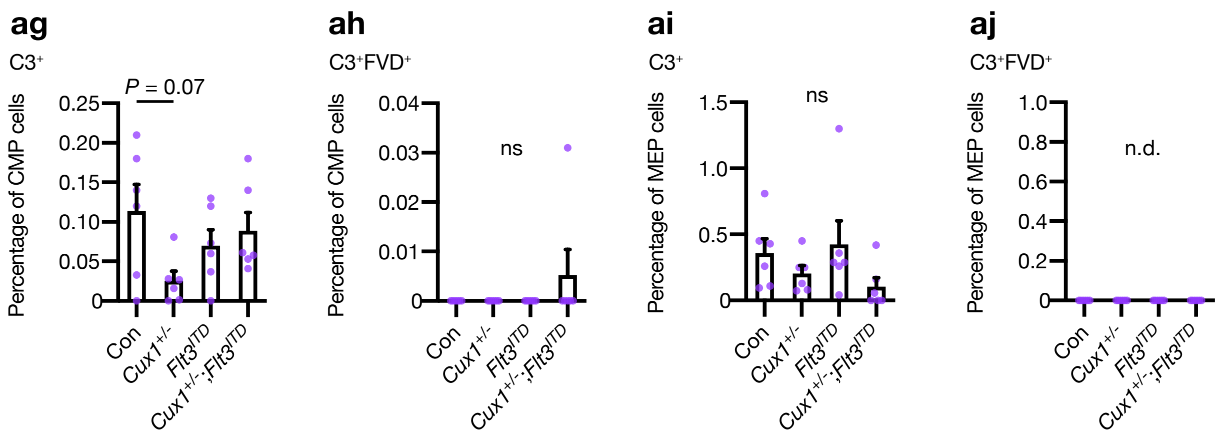

Supplementary Figure 4. Assessment of apoptosis defects in *Cux1$^{+/-}$* and *Cux1$^{+/-}$;Flt3^{ITD}* mice
(**a-d**) Plots showing percentage of early- (Annexin-V$^+$; AV$^+$) and late- (AV$^+$fixable viability dye$^+$ [FVD$^+$]) apoptotic cells in LT-HSC (a,b) and ST-HSC (c,d) in 10-12 week-old mice from control (Con), *Cux1$^{+/-}$*, *Flt3^{ITD}* and *Cux1$^{+/-}$;Flt3^{ITD}* genotypes (n = 6 per group).
(**e-h**) Plots showing percentage of early- (AV$^+$) and late- (AV$^+$FVD$^+$) apoptotic cells in CMP (e,f) and MEP (g,h) cells.
(**i-t**) Absolute numbers of LSK (i), LK (j,k), CMP (l,m), GMP (n,o), MEP (p,q), LT-HSC (r), ST-HSC (s) and MPP (t) early- (AV$^+$; green circles) and late- (AV$^+$FVD$^+$; blue circles) apoptotic cells in two leg bones from mice of the indicated genotypes.
(**u-ab**) Apoptosis in SLAM marker-stained LSK HSCs was assessed by staining for active caspase-3 (C3) and FVD to identify early (C3$^+$) and late- (C3$^+$FVD$^+$) apoptotic cells in LSK (u,v), MPP (w,x), LT-HSC (y,z) and ST-HSC (aa,ab). Percentages of early- and late-apoptotic cells in each compartment are shown.
(**ac-aj**) GMPs, CMPs and MEPs were identified by flow cytometry in LK MPs and apoptosis was assessed by staining for active caspase-3 (C3) and FVD to identify early (C3$^+$) and late- (C3$^+$FVD$^+$) apoptotic cells in LK (ac,ad), GMP (ae,af), CMP (ag,ah) and MEP (ai,aj). Percentages of early- and late-apoptotic cells in each compartment are shown. Each circle represents one mouse. Plots show mean + s.e.m; ns, not significant; n.d., not detected; one-way ANOVA with Tukey's test for multiple comparisons (a-aj).

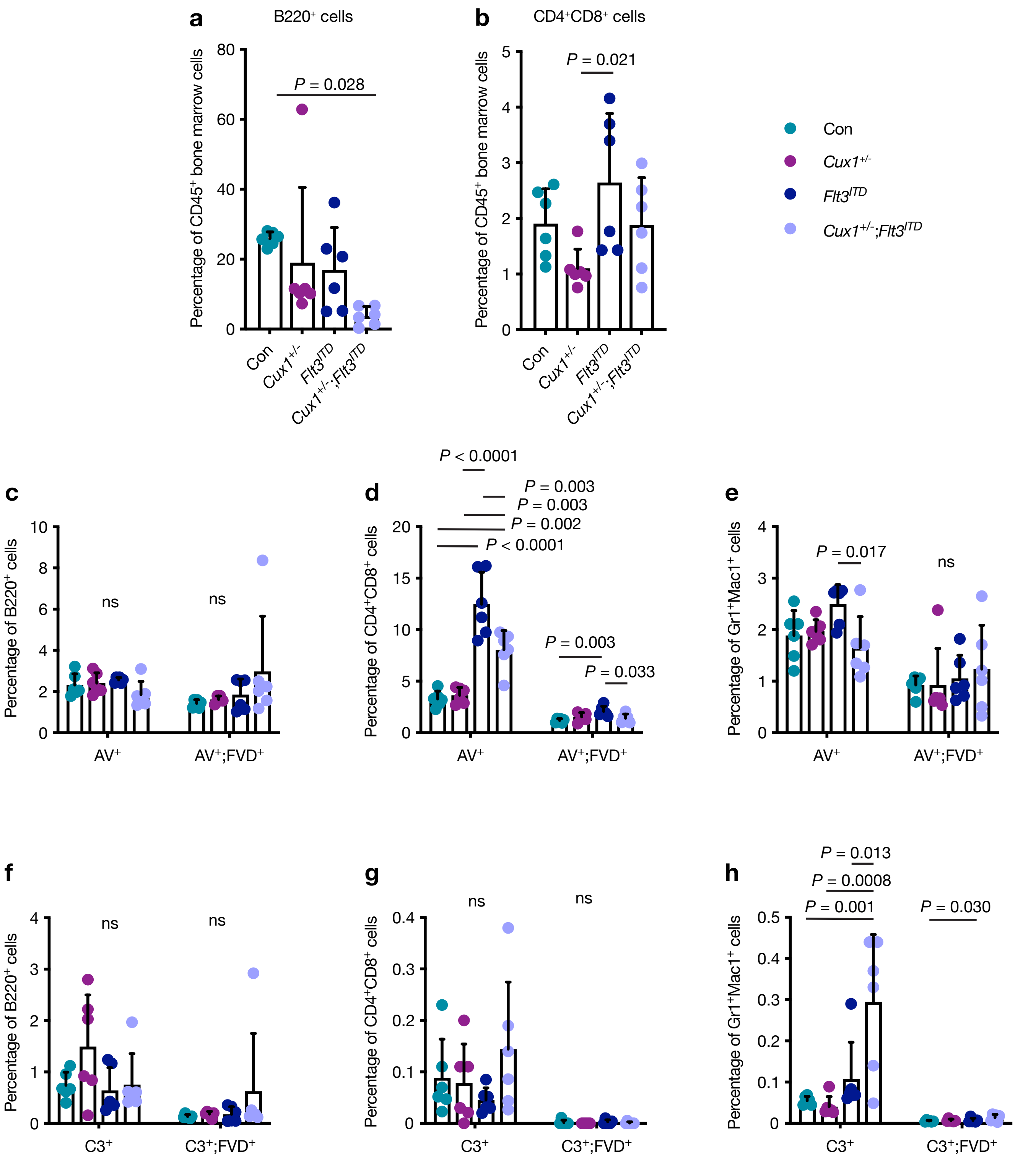

Supplementary Figure 5

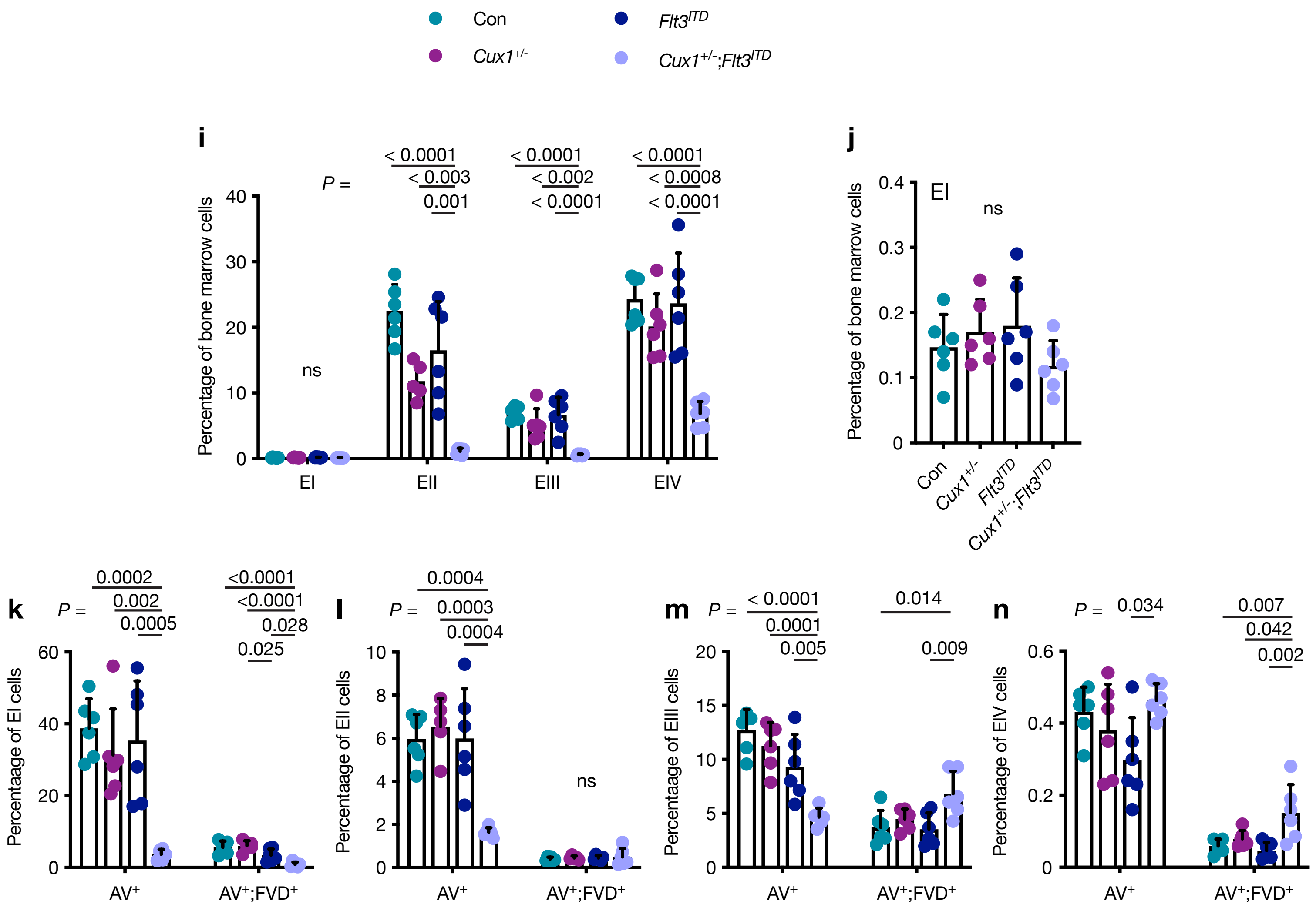

Supplementary Figure 5. Assessment of apoptosis defects in hematopoietic lineage-positive *Cux1*$^{+/-}$ and *Cux1*$^{+/-}$;*Flt3*^{ITD} cells

(**a,b**) Percentage of bone marrow B220^{+} B cells (a) and CD4^{+}CD8^{+} T cells (b) from 10-12 week-old control (Con), *Cux1*$^{+/-}$, *Flt3*^{ITD} and *Cux1*$^{+/-}$;*Flt3*^{ITD} mice (n = 6 per group).

(**c-e**) Plots showing percentage of early- (Annexin-V^{+}; AV^{+}) and late- (AV^{+}fixable viability dye^{+} [FVD^{+}]) apoptotic cells in bone marrow B220^{+} B cells (c), CD4^{+}CD8^{+} T cells (d) and Gr1^{+}Mac1^{+} (e) myeloid cells from mice assessed in (a,b).

(**f-h**) Plots showing percentage of early- (C3^{+}) and late- (C3^{+}FVD^{+}) apoptotic cells in B (f), T (g) and myeloid (h) cells from mice of the indicated genotypes.

(**i,j**) Plots showing percentage of bone marrrow stage EI-EIV erythroid precursor cells in mice of the indicated genotypes. An expanded view of EI cells is shown in (j).

(**k-n**) Plots showing percentage of early- (AV^{+}) and late- (AV^{+}FVD^{+}) apoptotic cells in each bone marrow erythroid precursor compartment. Each circle represents one mouse. Plots show mean + s.e.m.; ns, not significant; one-way ANOVA with Tukey's test for multiple comparisons (a-n).

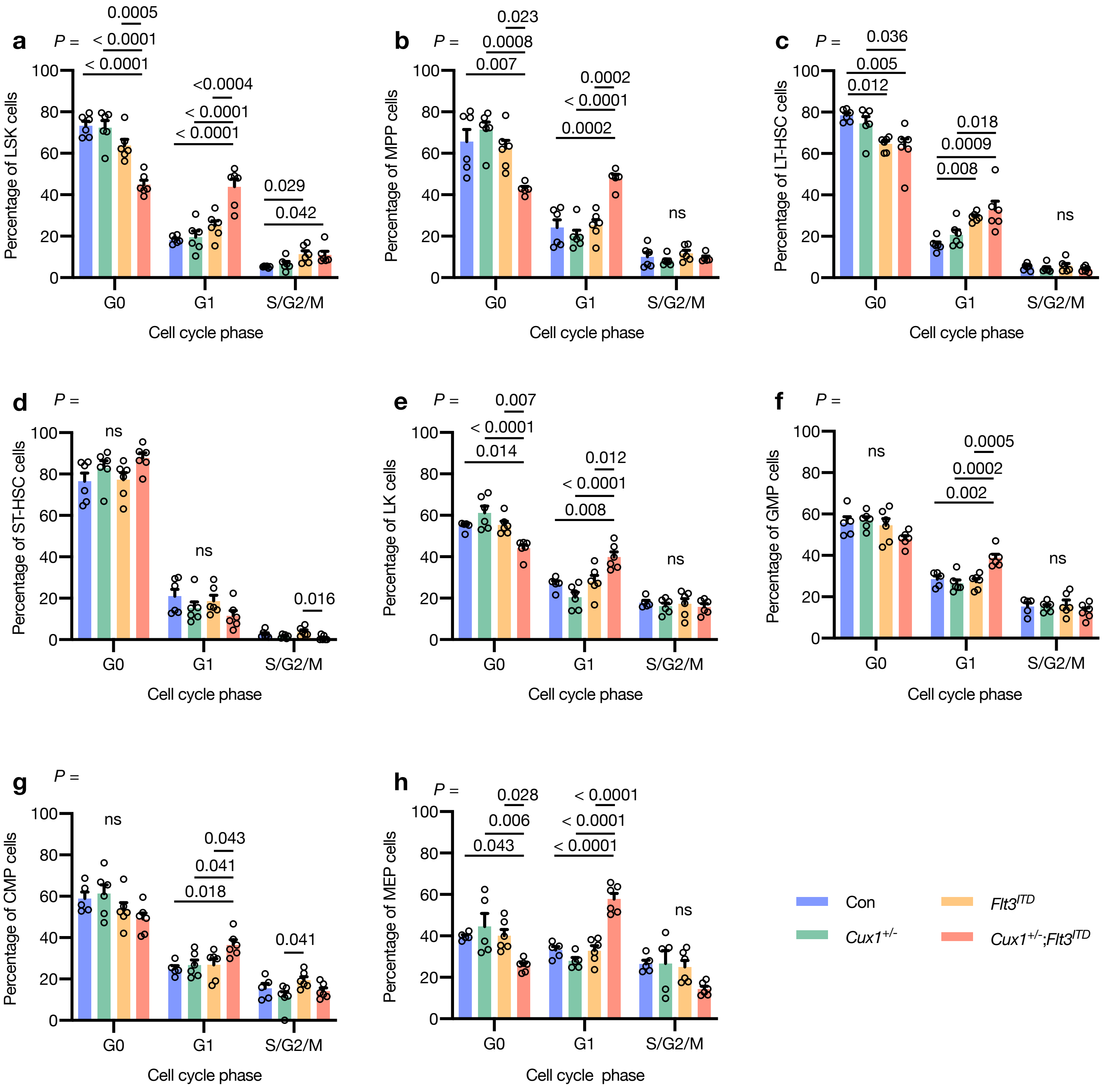

Supplementary Figure 6. Loss of cell cycle quiescence in *Cux1*$^{+/-}$;*Flt3*^{ITD} hematopoietic stem and progenitor cells

(**a**) Hematopoietic stem and progenitor cells were stained with antibodies to identify HSC or MP populations together with Ki-67 and DAPI in order to determine cell cycle phases of each compartment. Bar plot shows percentage of LSK cells in each cell cycle phase for five-six 10-12 week-old mice from the indicated genotypes.

(**b-d**) Cell cycle analysis for MPP (b), LT-HSC (c) and ST-HSC (d) compartments in mice from the indicated genotypes.

(**e-h**) Cell cycle analysis for LK (e), GMP (f), CMP (g) and MEP (h) compartments in mice from the indicated genotypes. Each circle represents one mouse. Plots show mean + s.e.m. One-way ANOVA with Tukey's test for multiple comparisons (a-h).

Supplementary Figure 6

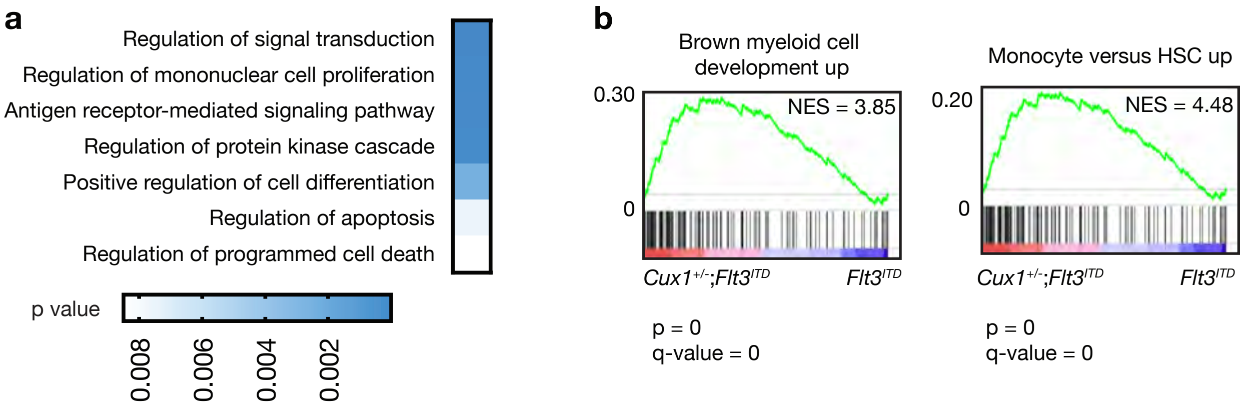

Supplementary Figure 7. Gene ontology and GSEA comparison of *Cux1$^{+/-}$;Flt3^{ITD}* and *Flt3^{ITD}* LSK cells

(**a**) Gene ontology analysis using DAVID showing pathways upregulated in *Cux1$^{+/-}$;Flt3^{ITD}* LSK cells compared with *Flt3^{ITD}* cells.
(**b**) GSEA of transcriptomes comparing *Cux1$^{+/-}$;Flt3^{ITD}* with *Flt3^{ITD}* LSK cells. *Cux1$^{+/-}$;Flt3^{ITD}* cells show enrichment of myeloid development and monocyte gene signatures. NES, normalized enrichment score; p, nominal p value; q-value, false discovery rate q-value, accounting for multiple comparisons.

Supplementary Figure 7

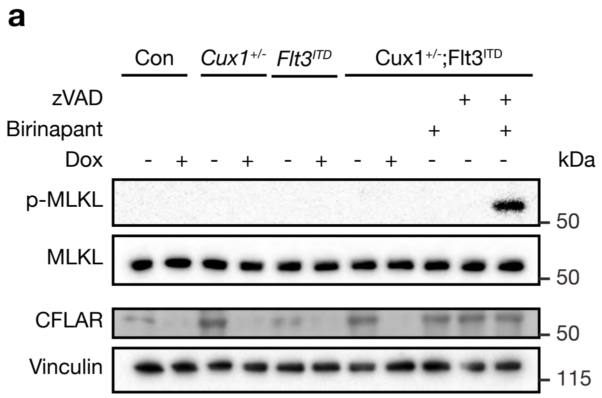

Supplementary Figure 8.
Absence of MLKL phosphorylation following Cflar depletion in Cux1$^{+/-}$Flt3^{ITD} cells.
(**a**) Hematopoietic cKit^{+} cells from control (Con), *Cux1$^{+/-}$*, *Flt3^{ITD}* and *Cux1$^{+/-}$;Flt3^{ITD}* mice were treated with doxycycline to induce Cflar knockdown or drug vehicle. Lysates were prepared after 48 h of treatment and used for immunoblotting to detect the indicated proteins. As a positive control for MLKL phosphorylation, *Cux1$^{+/-}$;Flt3^{ITD}* cKit^{+} cells were treated with birinapant (500 μM) and/or zVAD (10 μM) which in combination can induce necroptosis. Immunoblotting was conducted once.

Supplementary Figure 8

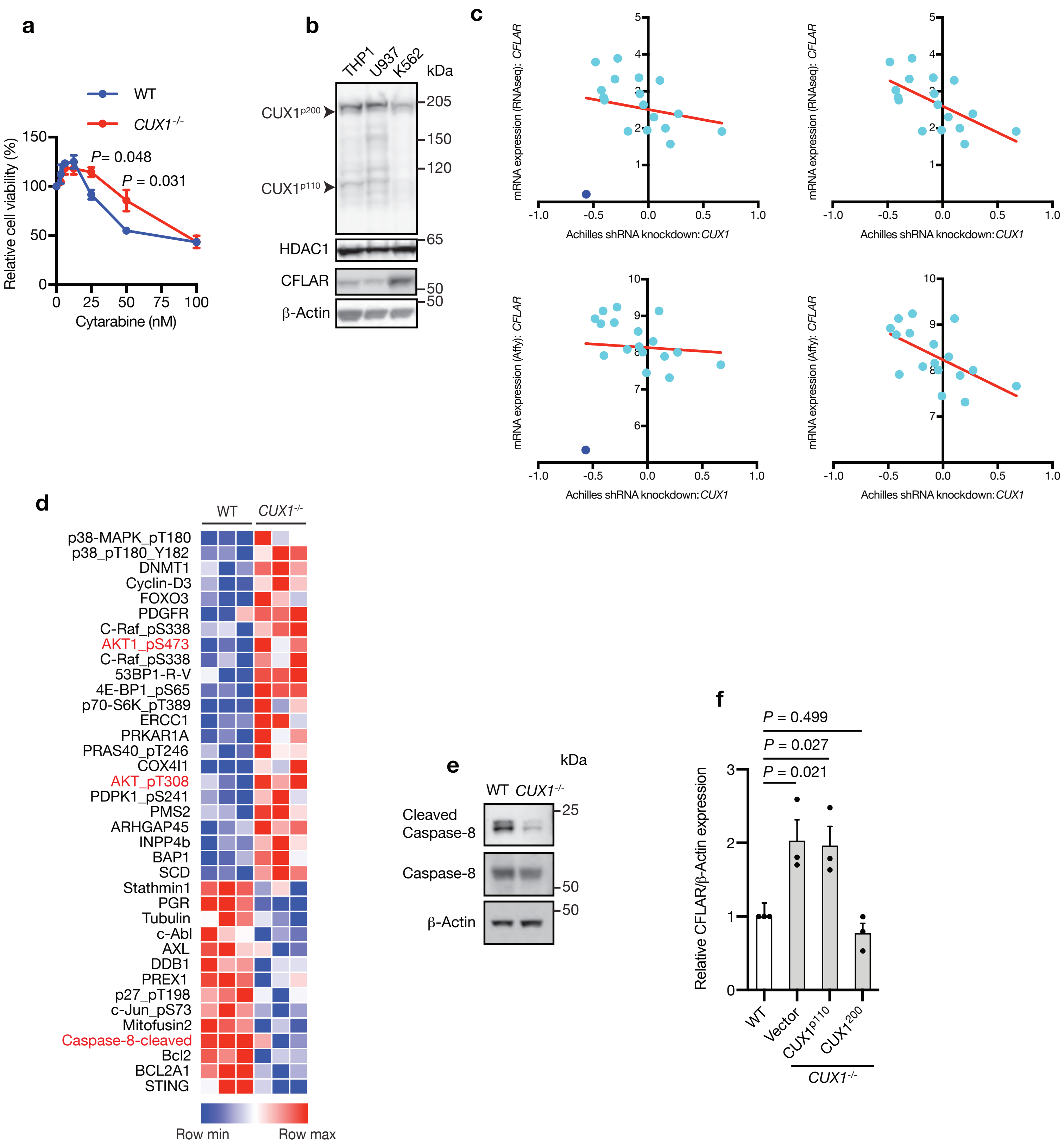

Supplementary Figure 9

Supplementary Figure 9

***CUX1* deficiency is associated with increased CFLAR levels and cytarabine resistance.**

(**a**) Cell viability dose-response curves of *CUX1*$^{-/-}$ and wild-type U937 cells exposed to cytarabine at the indicated concentrations for 48 h. Plot shows mean ± s.e.m. of three independent experiments.

(**b**) Immunoblot showing expression of indicated proteins in human AML cell lines. HDAC1 and β-Actin were used as loading controls. The experiment was performed once.

(**c**) Scatter plot of *CFLAR* levels as measured by RNA sequencing (top) or Affymetrix platforms (bottom) in 18 AML cell lines (left) following shRNA-mediated knockdown of *CUX1*. The right panels exclude the outlying SKNO1 cell line (blue circle). Data are from https://portals.broadinstitute.org/ccle.

(**d**) Heatmap showing $\log_2$ normalized protein levels determined by RPPA in wild-type and *CUX1*$^{-/-}$ U937 cells. Normalized linear values for each protein were row normalized (blue, row minimum; red, row maximum). Proteins with significantly different levels between wild-type and *CUX1*$^{-/-}$ U937 cells are shown ($P < 0.05$, two-tailed unpaired t-test). Proteins are ranked according to fold change of levels in *CUX1*$^{-/-}$ to wild-type cells. Phospho-AKT and cleaved caspase-8 are highlighted in red.

(**e**) Single immunoblot showing reduced expression of cleaved caspase-8 in *CUX1*$^{-/-}$ U937 cells compared with wild-type. β-Actin was used as a loading control.

(**f**) Quantification of CFLAR levels from Figure 8c normalized to β-Actin (n = 3 independent experiments). Expression of CUX1^{p200} suppresses CFLAR levels in *CUX1*$^{-/-}$ U937 cells. One-way ANOVA with Dunnett's test for multiple comparisons (f); two-tailed, unpaired t-test (a).

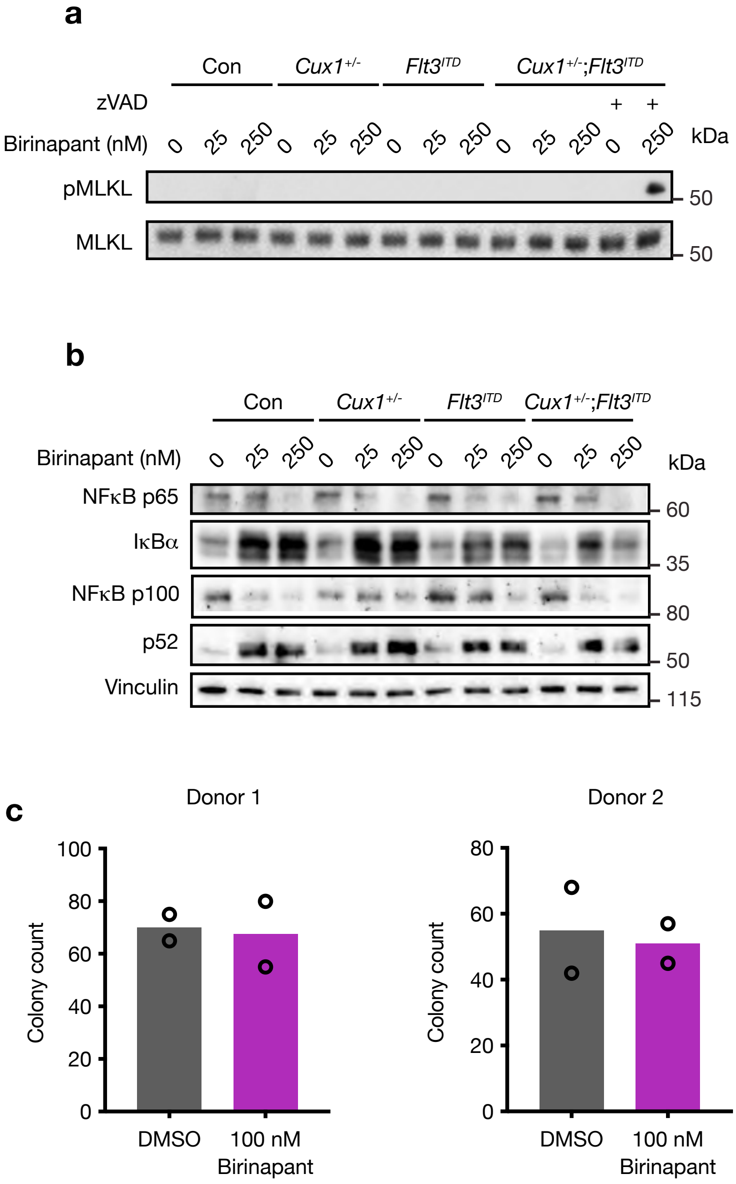

Supplementary Figure 10.

Impact of birinapant treatment on necroptosis, NFκB signaling and CD34⁺ cells.

(**a**) Immunoblotting shows lack of MLKL phosphorylation using c-Kit$^+$ cells lysates from control (Con), *Cux1*$^{+/-}$, *Flt3*^{ITD} and *Cux1*$^{+/-}$*Flt3*^{ITD} mice, which were treated with birinapant (0-250 nM) for 48 h. As a positive control for MLKL phosphorylation, *Cux1*$^{+/-}$*;Flt3*^{ITD} cKit$^+$ cells were treated with birinapant and zVAD (10 μM) which in combination can induce necroptosis in some contexts. The immunoblot was performed once.

(**b**) NFκB signaling was assessed by immunoblotting for pathway proteins using lysates from cells treated as in (a). Vinculin was used as a loading control. The blot was conducted once.

(**c**) 500 CD34$^+$ cells from two healthy donors were plated in methylcellulose media in duplicate with and without 100 nM birinapant. Colonies with more than 50 cells were scored 13 days later. Bar plot show mean.

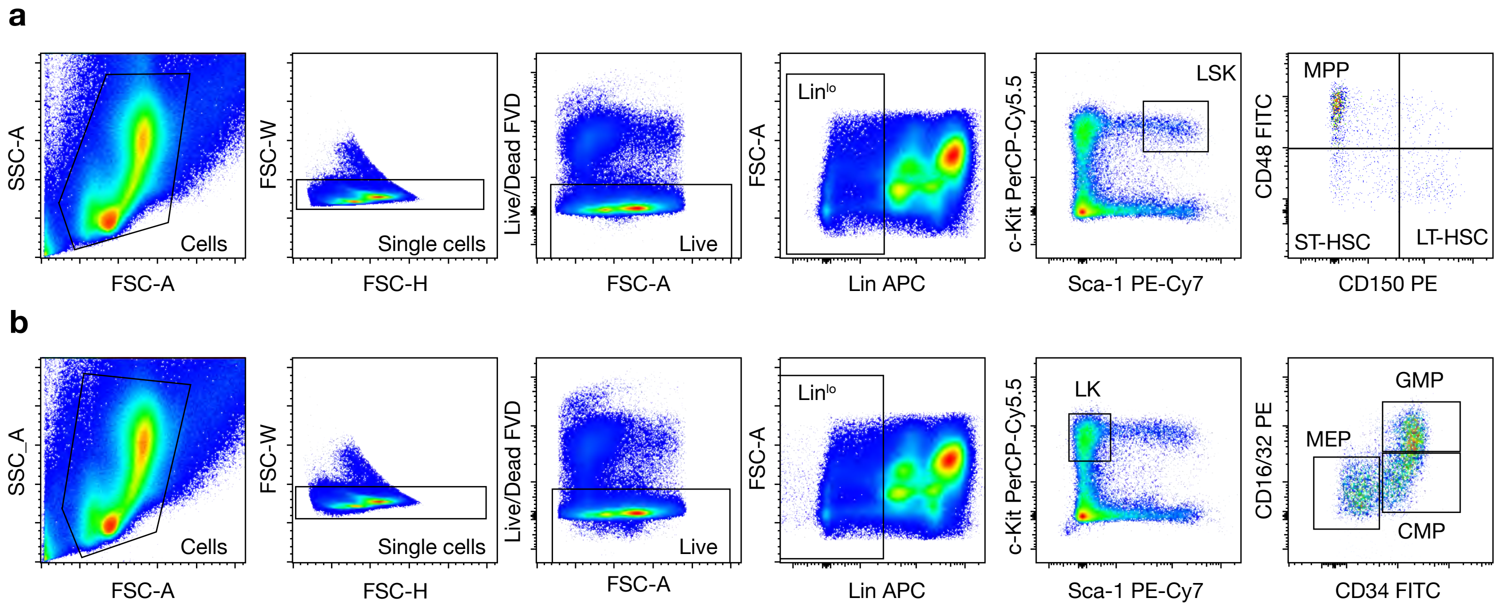

Supplementary Figure 11.
Flow cytometry-gating strategies.
(**a**) Strategy to identify hematopoietic LSK cells and SLAM populations within LSK compartment.
(**b**) Strategy to identify hematopoietic LK myeloid progenitor cells. Gating on LK cells allows identification of GMP, CMP and MEP populations.
