# Supplementary material for: Cut-like homeobox 1 (CUX1) tumor suppressor gene haploinsufficiency induces apoptosis evasion to sustain myeloid leukemia
Source: Nat Commun. 2021 Apr 30;12:2482. doi: 10.1038/s41467-021-22750-8 (PMC8087769; doi:10.1038/s41467-021-22750-8)
Supplement: Supplementary file 2 — Description of Additional Supplementary Files [file 41467_2021_22750_MOESM2_ESM.pdf]

Description of Additional Supplementary Files

File: Supplementary Data 1

Description: Table showing Bayes Factors and residual scores from genome-wide CRISPR/Cas9 screens in *CUX1*-knockout and *CUX1*-wild-type U937 cells.

File: Supplementary Data 2

Description: Table showing DAVID functional annotation of genes from CRISPR/Cas9 screen (Z score ≥3.5) that are potential genetic synthetic vulnerabilities in *CUX1*-knockout U937 cells.

File: Supplementary Data 3

Description: List of differentially expressed genes in LSK cells from $Cux1^{+/-}$;$Flt3^{ITD}$ versus $Flt3^{ITD}$ mice as determined by DESeq2 analysis. Genes with fold changes ≥ 1.5, $P_{adj} \leq 0.05$ are shown.

File: Supplementary Data 4

Description: Results of reverse phase protein array analysis of $CUX1^{-/-}$ (three clones, 1D, 1E and 2D) and wild-type (three clones, WT1-3) U937 cells. Normalized linear signal values for each protein are shown. *P* values were calculated using two-tailed, unpaired *t*-test and proteins with $P \leq 0.05$ are shown. The ratio of mean *CUX1*-knockout (KO) to mean wild-type (WT) level for each protein is also shown.

File: Supplementary Data 5

Description: Karyotype and fluorescence in-situ hybridization (FISH) characteristics of AML samples used in this study. FISH was used to detect common AML-associated copy number changes and translocations.

File: Supplementary Data 6

Description: List of oligonucleotides used in this study.
