# Supplementary material for: Cut-like homeobox 1 (CUX1) tumor suppressor gene haploinsufficiency induces apoptosis evasion to sustain myeloid leukemia
Source: Nat Commun. 2021 Apr 30;12:2482. doi: 10.1038/s41467-021-22750-8 (PMC8087769; doi:10.1038/s41467-021-22750-8)
Supplement: Supplementary file 4 — Reporting Summary [file 41467_2021_22750_MOESM4_ESM.pdf]

nature research

Corresponding author(s): Chi C. Wong

Last updated by author(s): 2021/03/06

Reporting Summary

Nature Research wishes to improve the reproducibility of the work that we publish. This form provides structure for consistency and transparency in reporting. For further information on Nature Research policies, see Authors & Referees and the Editorial Policy Checklist .

Statistics

For all statistical analyses, confirm that the following items are present in the figure legend, table legend, main text, or Methods section.

n/a	Confirmed	
☐	☒	The exact sample size (*n*) for each experimental group/condition, given as a discrete number and unit of measurement
☐	☒	A statement on whether measurements were taken from distinct samples or whether the same sample was measured repeatedly
☐	☒	The statistical test(s) used AND whether they are one- or two-sided<br>*Only common tests should be described solely by name; describe more complex techniques in the Methods section.*
☐	☒	A description of all covariates tested
☐	☒	A description of any assumptions or corrections, such as tests of normality and adjustment for multiple comparisons
☐	☒	A full description of the statistical parameters including central tendency (e.g. means) or other basic estimates (e.g. regression coefficient) AND variation (e.g. standard deviation) or associated estimates of uncertainty (e.g. confidence intervals)
☐	☒	For null hypothesis testing, the test statistic (e.g. *F*, *t*, *r*) with confidence intervals, effect sizes, degrees of freedom and *P* value noted<br>*Give P values as exact values whenever suitable.*
☒	☐	For Bayesian analysis, information on the choice of priors and Markov chain Monte Carlo settings
☒	☐	For hierarchical and complex designs, identification of the appropriate level for tests and full reporting of outcomes
☒	☐	Estimates of effect sizes (e.g. Cohen's *d*, Pearson's *r*), indicating how they were calculated

Our web collection on statistics for biologists contains articles on many of the points above.

Software and code

Policy information about availability of computer code

Data collection

Provide a description of all commercial, open source and custom code used to collect the data in this study, specifying the version used OR state that no software was used.

Data analysis

Flow cytometry data were analyzed using Flow Jo software (Tree Star, v10.0.8r1).
Micrographs: ImageJ (v1.51, NIH). Colony counting: ImageJ (v1.52b, NIH).
CRISPR/Cas9 data were analyzed using BAGEL (v. 0.9) to calculate Bayes Factors.
GraphPad Prism (v7 or v8) was used to plot graphs and to conduct statistical analyses: Two-tailed, unpaired t-tests or Mann-Whitney tests were performed for pairwise comparisons; log-rank (Mantel-Cox) tests were performed on survival curves to determine significant differences; and one-way or two-way ANOVA with Tukey's or Dunnett's tests were used for multiple group comparisons.
For mouse transcriptome data analysis:
STAR (v2.5.0), HTSeq (v.0.7.2), DESeq2 package (v1.14 or v.1.24.0).
GSEA v.4.0.3
DAVID v6.8
Software was not used for data collection.

For manuscripts utilizing custom algorithms or software that are central to the research but not yet described in published literature, software must be made available to editors/reviewers. We strongly encourage code deposition in a community repository (e.g. GitHub). See the Nature Research guidelines for submitting code & software for further information.

Data

Policy information about availability of data

All manuscripts must include a data availability statement. This statement should provide the following information, where applicable:

- Accession codes, unique identifiers, or web links for publicly available datasets
- A list of figures that have associated raw data
- A description of any restrictions on data availability

The data for this study have been deposited in the European Nucleotide Archive (ENA) at EMBL-EBI under accession number PRJEB32921 (https://www.ebi.ac.uk/ena/browser/view/PRJEB32921). Molecular Signatures Database v7.2 is available through GSEA website (https://www.gsea-msigdb.org/gsea/msigdb/index.jsp). The source data underlying Figures 1-8 and Supplementary Figures are provided as a Source Data file. All the other data supporting the findings of this study are available within the article and its supplementary information files and from the corresponding author upon reasonable request.

Field-specific reporting

Please select the one below that is the best fit for your research. If you are not sure, read the appropriate sections before making your selection.

☒ Life sciences ☐ Behavioural & social sciences ☐ Ecological, evolutionary & environmental sciences

For a reference copy of the document with all sections, see nature.com/documents/nr-reporting-summary-flat.pdf

Life sciences study design

All studies must disclose on these points even when the disclosure is negative.

Sample size	No mathematical or statistical methods were used to determine sample sizes for animal studies since the expected effect of the genetic manipulations on tumor phenotypes were not known beforehand.
Data exclusions	No data were excluded from the analyses.
Replication	The number of replication attempts for each experiment are detailed in the manuscript. Flow cytometry analysis for hematopoietic stem and progenitor compartments was performed on at least two separate occasions using mice from all assessed genotypes.
Randomization	Randomization was not applied to the animal studies; sex and age-matched animals were used for control and experimental arms.
Blinding	Blinding for animal studies was not employed in this the study as mouse genotypes needed to be determined prior to downstream experiments.

Reporting for specific materials, systems and methods

We require information from authors about some types of materials, experimental systems and methods used in many studies. Here, indicate whether each material, system or method listed is relevant to your study. If you are not sure if a list item applies to your research, read the appropriate section before selecting a response.

Materials & experimental systems

n/a	Involved in the study	
☐	☒	Antibodies
☐	☒	Eukaryotic cell lines
☒	☐	Palaeontology
☐	☒	Animals and other organisms
☒	☐	Human research participants
☒	☐	Clinical data

Methods

n/a	Involved in the study	
☒	☐	ChIP-seq
☐	☒	Flow cytometry
☒	☐	MRI-based neuroimaging

Antibodies

Antibodies used	Anti-myeloperoxidase antibody (1:100, ab9535, Abcam)<br>Antibodies to Gr1 (RB6-8C5, 108404, BioLegend), CD11b (M1/70, 101204, BioLegend), Ter119 (TER119, 116204, BioLegend), B220 (RA3-6B2, 103204, BioLegend), CD4 (RM4-5, 100508, BioLegend), CD8 (53-6.7, 100704, BioLegend) and CD3 (145-2C11, 100304, BioLegend) antibodies conjugated to biotin were used at 1:100.<br>c-Kit (2B8, 560557, BD Biosciences), Sca1 (D7, 558162, BD Biosciences), CD150 (TC15-12F12.2, 115904, BioLegend), CD48 (HM48-1, 103404,BioLegend), CD34 (RAM34, 11-0341-85, eBioscience), CD16/32 (93, 101308, BioLegend) were used at 1:100.<br>Streptavidin conjugated to APC (17-4317-82, eBiosciences), APC-Cy7 (405208, BioLegend) or eFluor 450 (48-4317-82, eBiosciences) was used at 1:200.

	Ki-67 (1:100, 16A8, 652406, BioLegend)<br>Anti-active caspase-3 Alexa Fluor 647 (1:50, 560626, BD Biosciences)<br>CD11b (ICRF44, 301310, BioLegend), CD14 (M5E2, 301804, BioLegend), CD34 (581, 343503, BioLegend) and CD33 (WM53, 303407, BioLegend) used at 1:100.<br>Cell Signaling Technology antibodies used at 1,1000: anti-HDAC1 (5356); anti-CFLAR (56343); anti-B-Actin (3700); anti-CASP8 (9746); mouse-specific anti-CASP8 (4927); anti-cleaved CASP8 (9496); mouse-specific anti-cleaved CASP8 (8592); anti-PARP (9542); anti-CASP3 (9662); anti-cleaved CASP3 (9661); anti-cleaved PARP (5625); anti-MLKL (37705); anti-p-MLKL S345 (37333); anti-XIAP (2042); anti-NFkB p65 (8242); anti-IkBa (4814); anti-NFkB p100/p52 (4882); and anti-vinculin (13901).<br>Other antibodies used at 1:1,000 dilution were: anti-CUX1 (ABE218, Millipore; 11733-1-AP, Proteintech); anti-myc (MA1-980, Invitrogen); anti-cIAP1/2 (MAB3400, Novus); and anti-Vinculin (SAB4200080, Sigma).<br>HRP-linked streptavidin (1:1,000, 3999) and secondary antibodies (1:2,000, anti-rabbit IgG, 7074; 1:2,000, anti-mouse IgG, 7076).
Validation	CUX1 and CFLAR antibodies were validated in this study by performing immunoblotting using lysates from CUX1 and/or CFLAR knockout or knockdown cells. Anti-MPO antibodies used for immunohistochemistry were verified by examining nuclear morphology positive-staining cells. Hematopoietic lineage, stem and progenitor cell staining using these antibodies have been described previously ref. 1, 2.<br>1. Kiel MJ, Yilmaz OH, Iwashita T, Yilmaz OH, Terhorst C, Morrison SJ. SLAM family receptors distinguish hematopoietic stem and progenitor cells and reveal endothelial niches for stem cells. Cell 121, 1109-1121 (2005).<br>2. Akashi K, Traver D, Miyamoto T, Weissman IL. A clonogenic common myeloid progenitor that gives rise to all myeloid lineages. Nature 404, 193-197 (2000).

Eukaryotic cell lines

Policy information about cell lines

Cell line source(s)	THP-1 cells were sourced from the Sanger Cell Lines Project (https://cancer.sanger.ac.uk/cell_lines). U937 cells originated from the European Collection of Authenticated Cell Cultures.
Authentication	Cells were authenticated by short tandem repeat genotyping.
Mycoplasma contamination	Cells were verified to be free from mycoplasma contamination.
Commonly misidentified lines<br>(See ICLAC register)	U937 have been reported to be contaminated in the past. U937 cells were used since they have myelomonocytic characteristics and are wild-type for CUX1.

Animals and other organisms

Policy information about studies involving animals; ARRIVE guidelines recommended for reporting animal research

Laboratory animals	Conditional Cux1 knockout mice, harboring loxP sites flanking Cux1 exons 15-18, were generated by a one-step CRISPR/Cas9 approach. Vav-iCre (008610, The Jackson Laboratory) and Flt3ITD (011112, The Jackson Laboratory) mice were also used in this study. Both male and female mice were used for long-term monitoring studies. For flow cytometry, 10 week-old mice were used unless otherwise indicated in the manuscript.
Wild animals	Wild animals were not used.
Field-collected samples	No field-collected samples were used.
Ethics oversight	All procedures were performed in accordance with UK Home Office regulations, UK Animals (Scientific Procedures) Act 1996 and with approval from the Animal Welfare Committee at the Wellcome Sanger Institute.

Note that full information on the approval of the study protocol must also be provided in the manuscript.

Flow Cytometry

Plots

Confirm that:

- [x] The axis labels state the marker and fluorochrome used (e.g. CD4-FITC).
- [x] The axis scales are clearly visible. Include numbers along axes only for bottom left plot of group (a 'group' is an analysis of identical markers).
- [x] All plots are contour plots with outliers or pseudocolor plots.
- [x] A numerical value for number of cells or percentage (with statistics) is provided.

Methodology

Sample preparation	Cell suspensions from mouse tissues were prepared by passing tissues through 70 μm cell strainers. Cells were depleted of red blood cells by treating with red cell lysis buffer were indicated.

Instrument	Flow cytometry data was acquired using an LSR II or Fortessa instrument (BD Biosciences). Cell sorting was done using a BD Influx machine (BD Biosciences).
Software	Flow cytometry data were analyzed using FlowJo software (v10.0.8r1, Tree Star).
Cell population abundance	Purity of sorted samples for RNA-seq library construction were verified by post-sort flow cytometry analysis.
Gating strategy	For HSC and MP analysis, cells were identified by FSC/SSC gating and doublets were excluded. Live cells were distinguished by DAPI or fixable viability dye exclusion and c-Kit/Sca1 gates were applied to the lineage-negative fraction to identify LK and LSK cells. CD34 and CD16/32 gates were applied to the LK gate to identify GMPs (Lin-Sca-1-cKit+CD34hiFcRII/IIIhi), CMPs (Lin-Sca-1-cKit+CD34hiFcRII/IIIlow) and MEPs (Lin-Sca-1-cKit+CD34lowFcRII/IIIlow). CD48 and CD150 gates were applied to the LSK gate to identify LT-HSCs (CD150+CD48-LSK), ST-HSCs (CD150-CD48-LSK) and MPPs (CD150-CD48+LSK).

☒ Tick this box to confirm that a figure exemplifying the gating strategy is provided in the Supplementary Information.
